# Multiscale synchronisation dynamics reveals the impact of an improvisatory approach to performance on music experience

**DOI:** 10.1038/s41598-025-90271-1

**Published:** 2025-03-24

**Authors:** Takayuki Nozawa, Madalina I. Sas, David Dolan, Hardik Rajpal, Fernando E. Rosas, Christopher Timmermann, Pedro A. M. Mediano, Keigo Honda, Shunnichi Amano, Yoshihiro Miyake, Henrik J. Jensen

**Affiliations:** 1 Tokyo Institute of Technology, Tokyo, Japan; 2 University of Toyama, Toyama, Japan; 3 Imperial College London, London, UK; 4 Guildhall School of Music and Drama, London, UK; 5 University of Sussex, Brighton, UK

**Keywords:** Multiscale coordination, Movement synchrony, Collective experience, Music performance, Classical improvisation, Psychology, Nonlinear phenomena, Social behaviour

## Abstract

Experiences of collective creative activities play an essential role in human societies, yet these experiences are particularly hard to capture, making their scientific study challenging. In a classical music concert-experiment performed by a string quartet, we contrast a *Let-go* performance mode, characterised by a more creative and improvisatory approach that encourages risk-taking and spontaneous expression, with a more *Strict* mode which requires adhering closely to the score, common in many Western classical music performance environments. We investigate the experience of audience members by analysing their subjective reports and movement patterns. Our results show that during performances in *Let-go* mode, movement synchronization was reduced between performers and audience members in shorter timescales, while the synchronization and its temporal variability were enhanced in longer timescales. Furthermore, these differences in the synchronization dynamics are predictive of changes in the audience’s perception of music. These results provide a first step towards the quantification of some of the fundamental aspects of collective music experiences. Specifically, the reported findings demonstrate the relevance of the often-neglected multiscale coordination between audiences and performers, and explain how this rich tapestry of physical behaviour is connected with the quality of the collective music experience.

## Introduction

Collective activities involving shared experiences are ubiquitous in human culture. They are believed to play crucial roles for strengthening social bonds, sense of group belonging, and social cohesion^1,2^. Empirical investigations have extensively explored the impact of interpersonal synchronization of physical activity on these social dynamics. Such synchronization is strongly associated with collective subjective experiences^3–5^. These include feelings of unity and perceived social bonding^6^, and the enhanced group experience in shared social and ritual celebrations^7,8^. Moreover, synchronization has been linked to positive objective outcomes such as occurrence of face-to-face communication and emergence of functional roles in various types of verbal and non-verbal interaction^9,10^.

Among collective activities, music making and listening occupies an important place in all known human societies^11,12^, and often reveals, even within unique, cultural-specific approaches, universal elements of expressing and perceiving emotional cues^13^. As a joint activity, ensemble music making requires high levels of empathy^13–15^, coordination, and synchrony^16^, which support the emergence of leadership^17^, improvisation^18,19^, and group states of flow^20,21^, and moreover, is known to engage audiences in a participatory, reciprocal relationship with the performers^22,23^.

Within musical praxis, musical improvisation is a very complex, creative and—when performed in an ensemble—highly social process, which requires years of training^24^. Improvisation has widespread appeal, as evidenced by its varied expressions across different cultures and musical genres^25,26^.

Yet, from the early 20th century until recently, the mainstream of Western classical music performance largely adhered to notation-based performance. Performers aimed to follow the score strictly and accurately, striving for the best and most expressive performance while avoiding spontaneous, improvisatory elements^27^.

In the last three decades, the concept of re-integrating a more improvisational approach to music performance is regaining attention in Western classical music research, teaching, and to some extent, practice. This can be seen, for example, in the creation, back in 2008, of the METRIC network: a project focusing on re-integrating improvisation in European conservatoires^28^. This project included 18 actively involved European conservatoires that are internationally renowned. Overall, compared to the last quarter of the previous century, there has been a vast increase in the volume of research focusing on the phenomenology, teaching and performance-related parameters of classical improvisation in Western art music. Importantly, several studies place the omission of improvisation from classical music performance and training under question, as this practice has been shown to enhance the musical experience of both performers and audiences^29,30^. However, this remains more of an exception rather than the norm in Western classical concert culture at large, unlike in several non-European music cultures such as Arabic and Indian art music.

We refer to the two performance modes described above as *Strict* and *Let-go*, respectively^30^. Namely, the *Strict* mode aims to follow the written score closely, avoiding any gesture not directly indicated by the written text. In contrast, *Let-go* mode actively incorporates a creative, ‘beyond the score’ approach^31^ to interpretation with real-time improvisatory concept that involves risk-taking and spontaneous expression^29,30^. *Strict* and *Let-go* performance modes are the two ends of a continuum, in which the Let-go end represents an improvisational state of mind. This continuum enables us to assess different degrees of strictness vs. improvisatory approach. Daniel Leech-Wilkinson’s reference to the “norms” (and the policing applied to ensure their maintenance)^32^ is similar to what we refer to as *Strict*, while the other end, *Let-go* or improvisational state of mind, belongs to what Wilkinson refers to as “... and how to escape them”^32^. For young performers, participating in international music competitions involves adhering to these norms, in addition to technical mastery. While some manage to keep a personal voice and narrative, many choose to avoid any risk-taking, aim for precision in executing the written score, and keep spontaneity to a minimum. Associating the strict mode of playing with international competitions came up during conversations with the musicians who participated in the concert experiment.

It is important to clarify what we mean by *Let-go*, and its place in the spectrum of improvisatory performance. Within the continuum of composition-improvisation used in many studies of improvisation^25^, the focus is very often on the dimension of “what”—the notes being played, or the musical contents of the piece being played. In our study, we broaden the focus to explore the level of “how” of performance, which encompasses a wide spectrum of performance dynamics. This includes the interaction between performers and the audience, as well as the performers’ real-time decisions and adaptations. In particular, *Let-go* mode involves a unique state of flow^20^ the performers find themselves in, and their phenomenology in the moment of performance. Contrary to the *Strict* mode in relation to performance, the “improvisational state of mind” exists within a flow-state characterised by risk-taking, more spontaneity and a more creative approach. The extra risk-taking and application of creativity does not necessarily have to result in changing any notes; it can be manifested in far-reaching changes—compared to the norms—of performance-related parameters: timing (tempi, rubati), dynamics (changes of loudness and how predictable or not these changes are), and timbre^30^.

In the context of Western classical music, coordination of physical movements between performers has been investigated^33,34^, yet previous studies have rarely studied whether and how physiological or movement synchrony occurs between music performers and their audiences, or the synchrony among listeners themselves. Recently, it has been shown that audiences synchronise on physiological signals such as heart and respiration rate in many situations of music-listening^35–39^. But regarding their physical motion, audiences to classical music are still mostly assumed to be passive and static, in contrast to audiences in other musical genres^40,41^.

In this paper, we challenge this assumption and explore the effect of adopting an improvisational approach to performance on the collective motion of seated audience members attending a classical chamber music concert. For this purpose, we developed a concert-experiment where two classical repertoire pieces were played twice, each in the *Strict* and *Let-go* performance modes, in order to compare and contrast between the two modes.

Albeit many psychological studies of music and improvisation tend to focus on short segments of a few measures being performed by many musicians^42,43^, the current experimental design allows us to address the improvisational character of performance and study its effects on the musical experience more comprehensively in an ecological, naturalistic manner.

During the experiment, we measured the spontaneous movement of the audience. Although maybe subtle, we expect their physical activity to be linked to their experience of the music, as well as to the movements of performers. Thus, we hypothesise the degree of movement synchrony in the audience reflect the way the audience perceive the different performance modes. Specifically, we hypothesise:

1. *Let-go* would be perceived by the audience as more innovative and improvisatory than *Strict* performances. This is in line with previous work^29,30^, and here we aim to confirm the results with a larger group;
2. *Let-go* would induce higher movement synchrony (between performers and audience and within the audience) than *Strict* performances. Furthermore, *Let-go* would also induce larger temporal variability in the degree of movement synchrony, due to the additional moments in which the audience face unpredictability or “surprise” in the music as it unfolds;
3. the effects on the audience’ perception and movement synchrony would be correlated. Specifically, higher movement synchrony and its temporal variability would be positively associated with the degree to which the audience perceives the music to be innovative and stimulating. We posit that such performances, by virtue of their novelty and engagement, capture the audience’s attention and foster a deeper connection with the performers and, indirectly, among the audience members themselves.

In addition to these main hypotheses, we also explore the role of psychological absorption in the audience’s perception of the music. Since psychological absorption is associated with the enjoyment of music^44^, we aim to test whether individual differences in the absorption trait may have any interactions with the effect of performance modes. Furthermore, we question whether visual perception of the performance is essential for the audience to appreciate *Let-go* performance mode. To test the possibility, we have blindfolded a subgroup of audience members, and investigate whether they respond differently to the two performance modes.

## Results

A classical concert-experiment showcasing a string quartet performance was arranged. During the event, the quartet presented each of the two repertoire pieces: Mozart’s string quartet *KV. 421 no. 15* (exposition of the first movement) and Haydn’s *Op. 76 no. 1* (third movement). Each piece was played twice: once in *Strict* mode and once in *Let-go* mode. For *Strict* mode, we instructed the quartet to aim for the level required in international competitions and perform to the best of their ability. In contrast, the performers were asked to adopt an improvisational state of mind when playing in *Let-go* mode^30^. Comparing *Let-go* with *Strict* performances of the same repertoire piece by the same performers allows us to experimentally manipulate collective musical experiences while controlling other factors such as piece characteristics and individual differences. The order of *Strict* and *Let-go* modes were exchanged between the pieces. The audience was not informed about the nature or the order of the two modes. The concert was attended by 42 audience members. Unlike other classical music studies, the audience consisted of younger listeners (most under 30) and a female majority. For more demographics data, see supplementary material Sec. A.1 . Questionnaire responses and movement data were collected in order to investigate how the two performance modes affect the audience’s experience. Details of the experimental design can be found in “Methods”.

### Performance ratings

As a first step in our analysis, to test our hypothesis 1, we investigated the subjective experience of audience members as reflected by questionnaire responses given after each pair of performances. Questionnaire scores were analysed via multilevel models that included experimental variables as fixed effects and participant IDs as random effects (see “Statistical tests”).

**Figure 1:**
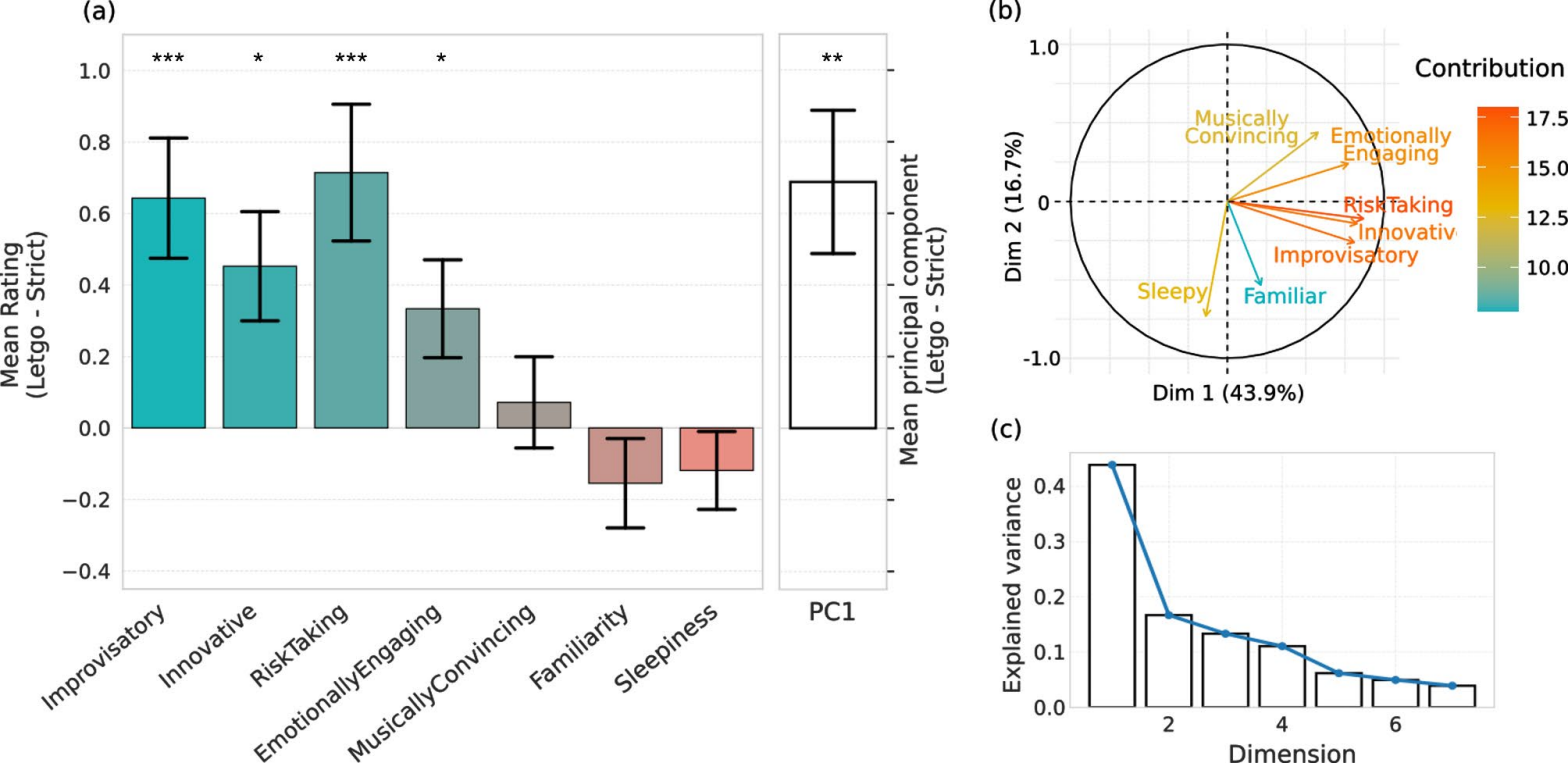
(**a**) Difference in the audience’s perception of the two repertoire works (by Mozart and Haydn) between the *Let-go* and *Strict* performance modes, including all 7 performance ratings and the first principal component (PC1). Error bars show standard error of the mean individual differences between the two modes of performance. Audience members perceived the *Let-go* mode of performance as significantly more Improvisatory, Innovative, and Risk-Taking. (**b**) Biplot of the contribution of the first two principal components towards the 7 audience ratings. (**c**) Scree plot showing the amount of explained variance by the 7 principal components.

Results in Fig. 1a reveal that the audience was receptive to the performance mode, rating the *Let-go* performances to be significantly more Improvisatory (standardized coefficient $\beta =0.41$, $t_{40}=3.78$, $p<0.001$), Innovative ($\beta =0.31$, $t_{40}=2.69$, $p=0.011$), Risk-taking ($\beta =0.44$, $t_{40}=3.75$, $p<0.001$), and Emotionally Engaging ($\beta =0.27$, $t_{40}=2.44$, $p=0.017$) than the *Strict*. This is in accordance with our hypothesis 1 and previous work^30^. In contrast, no significant differences were observed regarding how Musically Convincing both renditions were.

To quantify to what extent these ratings reflect either a unified factor or different aspects of the audience’s experience, we performed a principal component analysis (PCA) to evaluate how much variance in questionnaire scores can be explained as being part of a single factor. Results show that the first principal component (PC1)—mainly consisting of the Improvisatory, Innovative, Risk-taking, and Emotionally Engaging items—accounts for 43.9% of the variance (see Fig. 1b, c). Furthermore, the value of PC1 is significantly higher for the *Let-go* than the *Strict* mode ($\beta =0.39$, $t_{40}=3.12$, $p=0.003$; Fig. 1a right), supporting the conclusion that it captures a principal axis that differentiates between performance modes. See the supplementary Table S2 for the full statistical information.

To evaluate the potential effect of visual cues on the difference of experience between *Strict* and *Let-go*, 13 audience members were blindfolded. Incorporating the blindfolding factor in our multilevel models did not show a significant main effect of sight nor significant interactions with performance mode. A significant 3-way interaction was observed for the PC1, Improvisatory, and Risk-taking ratings. See the supplementary Table S2 for the full statistical information, and Fig. S2 for the visualization.

As an additional control, we investigated if the difference in the ratings between performance modes could be related to individual differences in psychological absorption in the audience members, as this trait has previously been linked to higher engagement with music^45^. Results show that absorption has a positive effect on the audience ratings in general, but not on differentiating the mode of performance, and no interaction with the mode of performance. We observe a significant effect on the audience’s Innovative ($$\beta =0.25$$, $$t_{39}=2.30$$, $$p = 0.027)$$ and Emotionally Engaging ($$\beta =0.30$$, $$t_{39}=3.14$$, $$p = 0.003$$) ratings, suggesting that higher absorption is indeed associated with a more positive emotional experience regardless of the mode of performance. It is insightful to observe the Musically Convincing rating and absorption are not significantly related ($$\beta =-0.01$$, $$t_{39}=-0.07,$$ $$p=0.947$$), and also that higher absorption subjects are likely to find the piece more Familiar ($$\beta =0.27$$, $$t_{39}=2.38$$, $$p=0.022$$). (See section B.2 in the Supplementary material for detailed results.)

### Movement synchrony

The second step in our analysis is to investigate the movement patterns of audience members, in particular the synchrony among listeners and with the performers and how they are affected by the performance modes (hypothesis 2). For this purpose, we carried out quantitative analyses using accelerometer data collected from the audience and performers.

#### Wavelet transform coherence analysis

**Figure 2:**
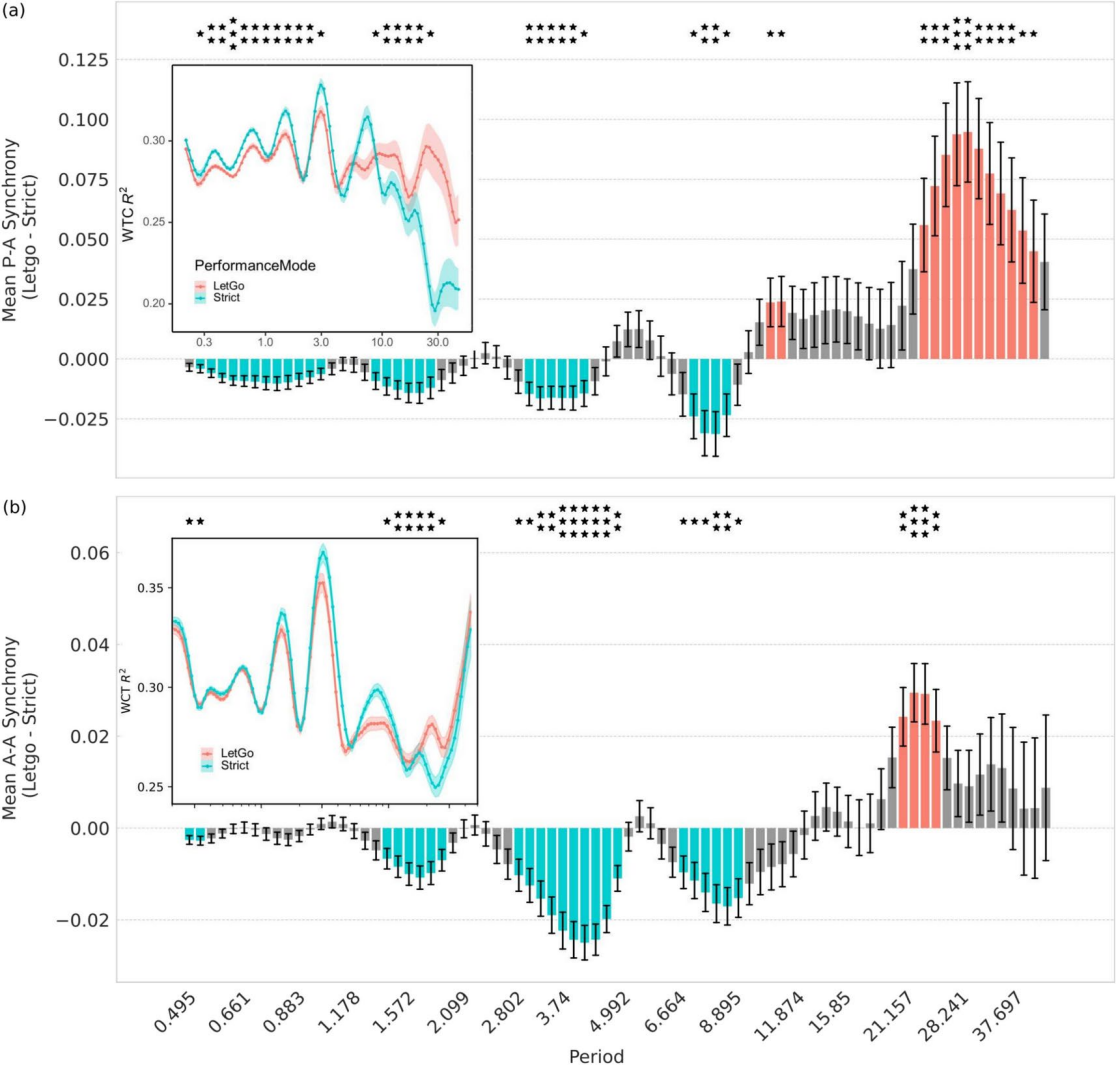
**(a)** Difference in performer–audience (P–A) synchrony at different timescales (Fourier periods) between the modes of performance, *Let-go* and *Strict*, for both repertoire works. Colours reveal the two synchrony regions: scales with negative differences between *Let-go* and *Strict* in blue (‘beat-sync’), and scales with positive differences between *Let-go* and *Strict* in red (‘music-sync’). **(a inset)** Mean P-A sync at different timescales for the two modes for the repertoire works. **(b)** Difference in mean audience–audience (A–A) sync between the two modes for both repertoire works. Colours reveal the two synchrony regions. **(b)** (inset) Mean A–A sync at different timescales for the two modes for the repertoire works. Error bars in the main plots and shared areas in the insets indicate standard error of the mean (SEM) over 42 listeners. Periods with significant differences between modes are marked by asterisks. *: p ${<}$ 0.05; **: p ${<}$ 0.01; ***: p ${<}$ 0.001; FDR-corrected.

We start with the degree of synchronisation across the entire spectrum of physical movement, considering the synchrony of movements between performers and audience (P–A sync) and also between audience members (A–A sync) over a wide range of timescales (Fourier periods). For this, we employ the wavelet transform coherence (WTC) on the time-frequency domain^46^, which has been widely used to evaluate interpersonal movement synchrony in various types of interactions, including in a musical context^18,47–51^.

When analysing synchrony at different timescales among audience members and between performers and audience, our results show that in both cases the audience exhibits higher synchrony in the *Strict* mode only at shorter timescales, while during the *Let-go* performances higher synchrony is seen at longer timescales (see Fig. 2).

Short timescales correspond to rhythmic elements of the piece as well as physiological signals such as breathing, and henceforth the synchrony that dominates in *Strict* can be referred to as **‘beat-sync’**. In contrast, the longer timescales, that dominate in *Let-go*, correspond to longer musical gestures related to higher-level semantics and musical expression^52^, which we therefore describe as **‘music-sync’**.

**Figure 3:**
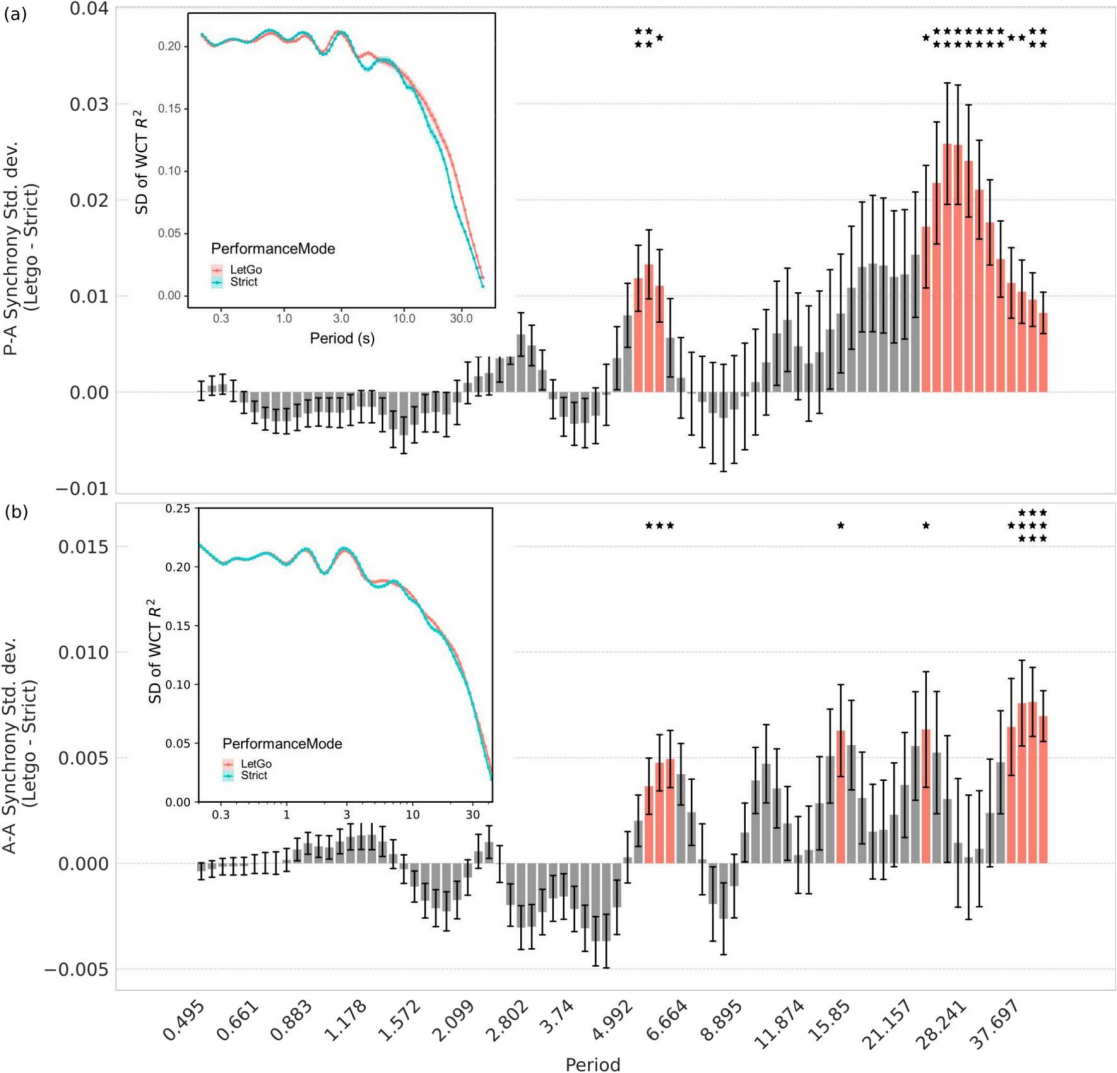
**(a)** Difference in the temporal variability (standard deviation) of performer-audience synchrony at different timescales between the two modes of performance, *Let-go* and *Strict*, for the two repertoire works. Red colour reveals the scales with significantly higher variability in *Let-go* than *Strict* mode (‘music-sync variability’). **(a inset)** Temporal variability of P-A sync at different timescales for the *Let-go* and *Strict* performances of the two repertoire works. **(b)** Difference in the temporal variability of A-A sync between the two modes of performance for both repertoire works. Red colour reveals the scales with significantly higher variability in *Let-go* than *Strict* mode (music-sync variability). **(b)** ( inset) Temporal variability of A-A sync at different timescales for the *Let-go* and *Strict* performances of the two repertoire works. Periods with significant differences between the two performance modes are marked by asterisks. *: p ${<}$ 0.05; **: p ${<}$ 0.01; ***: p ${<}$ 0.001; FDR-corrected.

In addition to the average P–A and A–A sync, we studied temporal variability of the P–A and A–A sync at each timescale. Results show that the audience exhibits significantly more variability of synchronisation at longer timescales during the *Let-go* performances (see Fig. 3). We refer to the temporal variability of the synchrony in these longer timescales as **‘music-sync variability’**. No significant differences were observed at shorter timescales.

Additional analyses showed no effects of blindfolding on the different types of synchrony and no significant interaction between visibility and performance mode. (See Section C.4 in supplementary material for details).

#### Breathing-related pattern analysis

One of the primary drivers of the beat-sync, especially at the period range of 3-5 s, is assumed to be the audiences’ respiration^53^. In order to deepen our understanding of the nature of this breathing-related component, we conducted further analysis on breathing-related patterns extracted from the accelerometer signals of each audience member, using alternative measures to complement the WTC analysis above.

**Figure 4:**
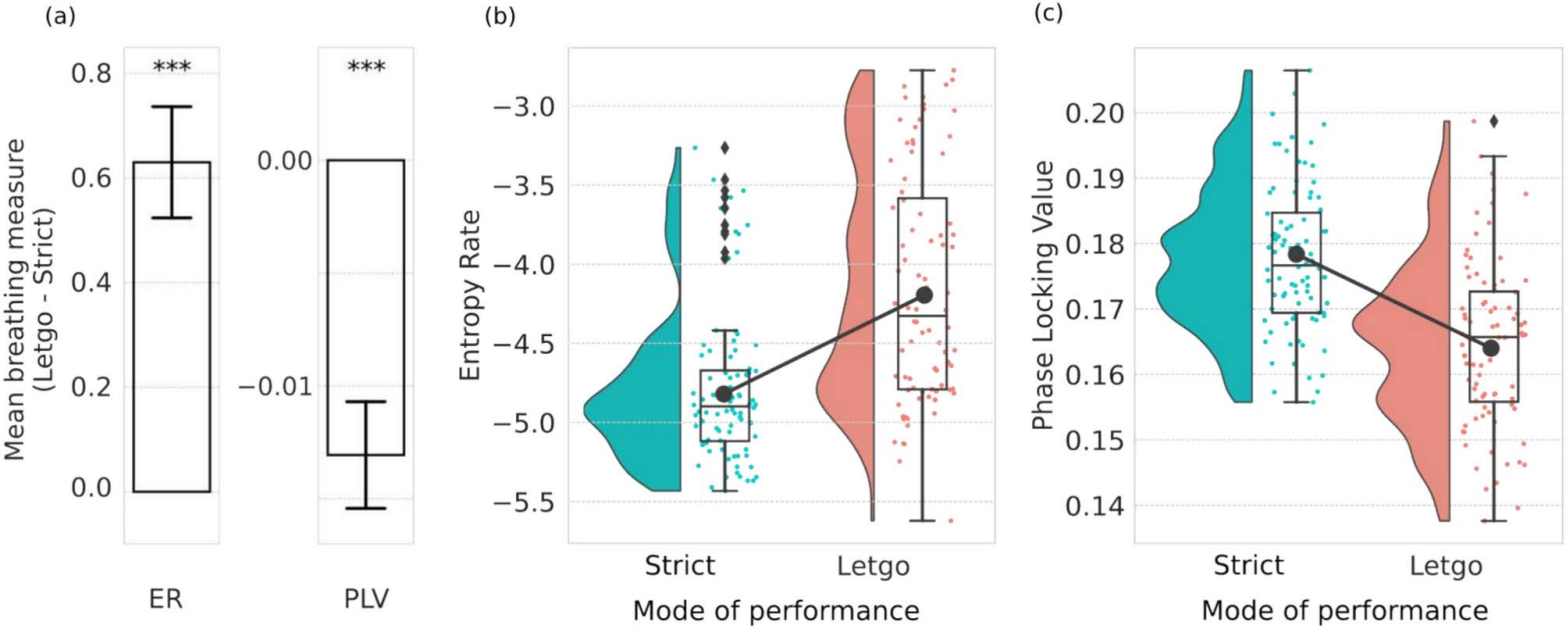
Comparison of breathing-related patterns between the two modes of performance combined over the 2 repertoire pieces. (**a**) ER of individuals’ breath-related signal is higher on average in *Let-go* ($\beta =0.72$, $t_{40}=5.89$, $p {<} 0.001$), while PLV is lower ($\beta =-0.51$, $t_{40}=-4.98$, $p {<} 0.001$). (**b**) Distribution of individual ER of breathing for the two modes. ER is higher in *Let-go*, showing increased variability. (**c**) Distribution of PLV between all pairs of subjects. On average PLV is higher in strict, corresponding to beat-sync.

We first investigated the diversity of breathing-related patterns exhibited by each individual by calculating their entropy rate (ER), which is a well-established information-theoretic metric of pattern diversity^54^. Results reveal an increase in ER of breathing-related patterns during the *Let-go* performance ($\beta =0.72$, $t_{40}=5.89$, $p {<} 0.001$, see Fig. 4), suggesting increased variability, which has been related to increased arousal with positive valence^55^.

By studying the level of synchrony between the breathing-related patterns of pairs of audience members via the phase locking value (PLV)^56^, we observe a significantly higher degree of synchrony in *Strict* than *Let-go* ($\beta =-0.51$, $t_{40}=-4.98$, $p {<} 0.001$). We also investigated the presence of higher-order synchronisation, namely at the level of triplets instead of pairs, among the audience members, but did not find significant differences (see Section C3 in the supplementary material for these results). The larger pairwise synchrony observed in *Strict*, which is contrary to our hypothesis 2, can be interpreted as arising from the more regular rhythms that characterise this performance mode and can be linked to the higher synchrony in beat-sync and the higher regularity of tempo in the *Strict* mode.

### Correlation between ratings and synchrony

As a final step in our investigation, we studied whether the differences in synchrony found in the previous section were predictive of the subjective experience as reported in the questionnaire ratings. For this, we built multilevel models using the various questionnaire items as dependent variables, and mean synchrony (either beat- or music-sync) or music-sync variability as independent variables, while accounting for subject ID using a random intercept (see “Methods” for details).

Results show that higher music-sync variability is most significantly associated with higher subjective scores (see Table 1), in particular the PC1 and items of the audience ratings that constitute perceived innovativeness of the performance. In contrast, increases in the average beat-sync were negatively associated with PC1 and the Improvisatory and Risk-taking ratings. This indicates that higher synchrony in this timescale was linked to lower audience perception of the performance as Improvisatory. The significant association between beat-sync and subjective scores were observed only in P-A sync and not in A-A sync. Changes in average music-sync between audience members or musicians were not associated with differences in the subjective scores.

**Table 1:** Association between audience ratings and mean movement synchrony in shorter and longer timescales, and the temporal variability of synchrony in the longer timescale. Values in the “*r*” columns represent correlation coefficients, which indicate the strength and direction of the associations. Values in the “$t_{125}$” columns represent *t* test statistics with 125 degrees of freedom, which are obtained by the multilevel models. Significant associations with $p<0.05$ are marked by bold face.

	**Beat-**			**Music-**			**Music-sync**		
	**sync**			**sync**			**variability**		
**Rating**	*r*	$t_{125}$	*p*	*r*	$t_{125}$	*p*	*r*	$t_{125}$	*p*
**P-A sync**									
PC1	**−0.18**	**−2.04**	**0.043**	0.10	1.13	0.260	**0.24**	**2.82**	**0.006**
Improvisatory	**−0.18**	**−2.03**	**0.044**	0.10	1.14	0.256	**0.21**	**2.36**	**0.020**
Innovative	−0.13	−1.42	0.158	0.07	0.75	0.453	**0.25**	**2.86**	**0.005**
RiskTaking	**−0.28**	**−3.21**	**0.002**	0.14	1.52	0.130	**0.25**	**2.93**	**0.004**
Engaging	−0.14	−1.55	0.124	0.08	0.94	0.347	0.09	0.97	0.336
Convincing	0.03	0.32	0.747	−0.02	−0.23	0.821	0.16	1.81	0.072
Familiar	0.08	0.95	0.344	−0.10	−1.17	0.245	−0.15	−1.66	0.099
Sleepy	0.12	1.40	0.164	−0.09	−1.05	0.295	−0.15	−1.71	0.090
**A-A sync**									
PC1	−0.08	−0.85	0.396	0.06	0.64	0.525	**0.29**	**3.43**	**<0.001**
Improvisatory	−0.14	−1.58	0.116	0.02	0.28	0.782	**0.34**	**3.98**	**<0.001**
Innovative	0.09	−1.02	0.307	0.11	1.28	0.202	**0.29**	**3.35**	**0.001**
RiskTaking	−0.16	−1.82	0.071	0.12	1.31	0.191	**0.29**	**3.41**	**<0.001**
Engaging	0.00	−0.04	0.965	0.00	−0.05	0.958	0.05	0.55	0.586
Convincing	0.12	1.36	0.175	0.01	0.12	0.906	**0.23**	**2.63**	**0.010**
Familiar	0.04	0.47	0.637	0.07	0.79	0.432	−0.02	−0.24	0.809
Sleepy	0.08	0.88	0.383	**0.21**	**2.37**	**0.019**	−0.16	−1.85	0.067

Overall, this suggests that having more dynamic synchrony at the scale of the musical gestures related to higher-level semantics and musical expression is associated with the distinctive experience provided by the *Let-go* performance, while having higher synchrony with musicians in the beat-sync scale is more characteristic of the less improvisatory experience.

It is worth noting that when focusing solely on Haydn’s composition (which musicians regarded as more successfully differentiated between *Let-go* and *Strict* modes), more statistically significant associations between mean beat-sync, mean music-sync, and music-sync variability with the ratings are observed, as shown in Table S4 in the supplementary material.

### Music performance analysis

Assuming that music serves as the primary driver of the collective behaviour observed in the audience, our findings on movement synchrony and the audience’s experience can be better contextualised by examining specific attributes of the musical performance. For this purpose, we conducted exploratory analyses based on the performers’ subjective reports, listening tests and quantitative performance analysis of the recordings in Sonic Visualiser^57^. See Methods for more details.

A brief study of the piece’s dynamics, by using the loudness curve reveals both *Let-go* performances had a larger dynamic range and showed higher pattern complexity, which is in line with a higher level of unexpectedness in terms of performed expressive gestures and phrasing, and also in accordance to subjective reports by the musicians. Moreover, we observe that the results related to pattern complexity of the loudness curves are consistent with mode of performance, with higher values in the *Let-go* performance mode.

Spectral entropy, on the other hand, which relates to the complexity of timbre, seems more consistent with the subjective reports of the musicians themselves, who maintained the modes of performance were not as clearly differentiated when performing Mozart’s quartet. (See Table 2 as well as *Limitations* below).

**Table 2:** Quantitative comparison between the modes of performance using tools from music performance analysis. Results in bold show consistent differences between the two modes in both pieces. *LUFS* loudness units relative to full scale.

	Mozart (*Strict*)	Mozart (*Let-go*)	Haydn (*Strict*)	Haydn (*Let-go*)
Tempo variability (s)	**0.083**	**0.088**	**0.175**	**0.227**
Mean Loudness (LUFS)	**−42.99**	**−42.19**	**−45.23**	**−44.16**
Std. Dev. Loudness (LUFS)	6.62	6.74	8.23	7.48
Loudness pattern complexity	**1100**	**1106**	**1419**	**1442**
Spectral entropy	15.57	15.42	16.22	16.36

Finally, we studied the tempi of the performances using graphs of tempo curves generated in Sonic Visualiser. For each performance, the musicians have established a beat of rhythmic reference based on the tempo, time signature and mode of performance (see Methods).

When analysing tempi curves of the peformance of Mozart’s quartet, we related to a crochet (1/4) as a beat of rhythmic reference. Measurements revealed that the average duration of a crochet in the *Strict* mode is 0.74s, and 0.744s in the *Let-go* mode. This makes the mean duration of a half-bar approximately 1.5 seconds, and the mean duration of a bar approximately 3 seconds. When analysing Haydn’s minuet performance’s tempi curve, we measure the tempo in relation to one whole bar of 3/4 as beat of rhythmic reference. This movement comprises three sections, *minuet-trio-minuet*, each with slightly different tempo, so we computed the average bar duration for each section. The average durations of each bar of three crochets is 0.6, 0.885, 0.551 seconds in *Strict*, and 0.631, 0.958, 0.585 seconds in *Let-go*, which all match to timescales included in beat-sync.

At times, the musicians created one uninterrupted gesture made of two or four bars in the case of Haydn’s minuet and a half-bar, one bar or sometimes two bars in the case of Mozart’s quartet. The duration of such musical gestures would range between 1 and 4 seconds. Therefore, in both the Haydn and Mozart performances, the mean duration of beats of rhythmic reference and musical gestures comprising of 2 or 4 such beats correspond to timescales within beat-sync. Moreover, timescales around 0.8, 1.5, and 3 seconds show significant peaks in synchrony and this tempo analysis suggests they may be related to the beat of rhythmic reference. These beat-sync peaks are higher during the *Strict* performances, where the musicians follow a more even and rigid rhythmic pulse.

On average, tempi were slightly slower in *Let-go* performance modes, and more importantly, we found tempo variability is consistently higher in Let-go than in *Strict* performance mode (See Table 2). The differences in synchrony scales are also supported by the difference in tempo variability in *Strict* as compared to *Let-go*. This result agrees with the musicians’ observation that the *Strict* performance mode is characterised by more rigidly even short-term beats and higher synchrony at a single, short-term beat level. It is also related to the fact that the audience may find these more rigidly even beats more predictable, likely causing the higher movement synchrony in short beat-sync timescales during *Strict* performances.

To explore the longer, music-sync timescales emergent in the movement synchrony, it is important to distinguish between the short-term metronomic beat—such as quavers or crochets, characterised by evenness between notes—and a broader structural pulse associated with longer, more variable durations. The latter is a deeper structural pulse which often governs longer musical phrases and facilitates more significant expressive potential. During the *Let-go* performance mode, in which an improvisational state of mind is applied, the length of expressive gestures marked by the deeper structural pulse can change according to the performer’s expressive intentions. Therefore, gestures and phrases may have longer durations. (We would like to note that musicians in general, not only conductors, often refer to the specific beat they focus on while performing as a *conducting beat*. This is often the beat of rhythmic reference explained above. However, in the *Let-go* performance mode, the deeper structural pulse sometimes functions as the *conducting beat*.) For example, in both performances of Mozart’s quartet, a 4-bar phrase would last on average 10-12 seconds, which is within the music-sync timescale for P-A sync. This 4-bar phrase is written in a classical-style, symmetric periodic phrase structure, which acts as a “call and response” and would be governed by the deeper pulse of half bar or whole bar rather than the metronomic crochet or quaver pulse. Longer durations in music-sync can be associated with even longer phrasing structures.

While listening to the performance recordings, the performers found that arriving together at the phrases’ goal points was more convincing in the *Let-go* performance mode, despite spontaneous deviations from some of the score’s instructions in terms of timing, dynamics, and at times actual notes (improvised elaborated repeats or cadential moments for example). Those changes represent an element of unknown and a higher level of risk-taking, which makes the stronger cohesion in phrasing far from obvious, and supports the higher music-sync and music-sync variability during *Let-go*.

## Discussion

This study investigated how the improvisational approach to performance, termed the *Let-go* mode, impacts the audience’s collective experience in comparison to the *Strict* performance mode, which represents the mainstream approach in Western classical music performance. The analysis focused on three aspects: (1) the audience’s ratings for the experiential part; (2) movement synchrony between the performers and audience, and among audience members, for the movement part; and (3) the relationship between the ratings and synchrony to integrate the experiential and movement aspects. We also conducted additional follow-up analyses to deepen our understanding of the mechanisms and factors influencing the impact of the improvisatory approach.

Consistently with previous studies^30^, audience rated *Let-go* performance higher than *Strict* counterparts in multiple experiential dimensions, suggesting that the experiment was successful in inducing differentiated musical experiences on the audience. The result supports our hypothesis 1. In particular, the audience perceived the higher Improvisatory, Innovative and Risk-Taking character of *Let-go* performances, while considering both performances as Musically Convincing. Additional analyses show no effects of blindfolding on ratings, suggesting that the music itself—rather than visual cues—acted as a driver for the collective subjective experience. Moreover, results show that performance ratings are also related to the psychological trait of absorption, but this does not explain away the effect of the performance mode. Absorption has been previously linked to the enjoyment of music^58^, yet it does not seem to affect collective engagement in the *Let-go* performance.

To explore the relationship between qualitative aspects of musical performance and audiences’ experience, we also gathered subjective accounts of the performance from the musicians. All members of the quartet recognised that during the first pair of performances (of Mozart’s piece), the discomfort they felt initially due to the experimental equipment affected their state of flow, causing their first performance to be more rigid than intended for a *Let-go* performance. In the second pair of performances (of Haydn’s piece), they felt more together and more emotionally connected during *Let-go*, and also recognised many more moments of significant emotional expression in the *Let-go* mode.

Interestingly, looking into the effect of compositions on the audience’s ratings, we find that the differences in PC1 and the Improvisatory, Innovative and Risk-taking ratings between the two performances of the piece by Mozart are weaker than those for the piece by Haydn, as revealed by significant interactions between the performance mode and composition factors by the multilevel models (Improvisatory: $\beta =-0.67$, $t_{40}=-3.67$, $p<0.001$; Innovative: $\beta =-0.59$, $t_{40}=-3.60$, $p<0.001$; Risk-taking: $\beta =-0.62$, $t_{40}=-2.97$, $p=0.005$; see supplementary Section B.1 for details). These results indicate that the audience perceived the differences between the *Let-go* and *Strict* performances of Haydn’s composition, but they were not as sensitive to the difference between *Let-go* and *Strict* performances of Mozart’s composition. Importantly, this is in accordance with the musicians’ subjective reports on their own performance.

Regarding our hypothesis 2, the results reveal that an improvisatory approach to performance affects movement synchrony in the audience in opposite directions, depending on the timescale. In effect, *Let-go* performances reduce synchrony comparing with *Strict* in shorter timescales, while they enhance synchrony on longer timescales. Short timescales can be associated with the rhythmic pulse and physiological responses to it, which are clearer in the Strict rendition of the music, and longer timescales with longer musical macrostructures and musical phrases^52^.

Our findings, therefore, suggest that collective music experience is embodied in a multiscale adaptive interaction between the performers and audiences, with these spanning a longer temporal horizon in *Let-go* renditions than in *Strict* ones. Similar time-scale dependency of the movement synchrony has also been observed in different forms of social interactions, including collaborative team problem solving^50^ and joke telling^47^.

It is worth noticing that the fact that synchrony was observed both for blindfolded and sighted audience members suggest that, in terms of mechanisms, audience modulated their movement synchrony with the performers mainly via auditory rather than visual information, which is in line with previous results^30^. This suggests, in turn, that performance-to-audience synchronisation was primary, and that audience-to-audience synchronisation emerged mainly indirectly, mediated by the former interactions—rather than by the direct interaction between audience members.

Our analysis of synchrony in movement patterns was not restricted to the average degree of synchrony, but also considered the variance of synchrony during the performance. Results show that *Let-go* performances increase the temporal variability of synchronisation on longer timescales. Combined with the results of the average sync, this means that the *Let-go* performances increased longer-timescale synchrony at specific timings rather than evenly throughout the performances. In other words, it enhanced the dynamical transition between a convergent (in-sync) phase, where the movements of audience members and performers are well-coordinated, and a divergent (out-of-sync) phase, where their movements are less coordinated. From a dynamical systems perspective, this temporal variability in synchrony could be interpreted in terms of meta-stability, which captures the temporal dynamics between high-synchrony phase where the involving elements are coordinated and integrated into quasi-stable low-dimensional states, and low-synchrony phase where the elements are segregated leading to transient uncoordinated states^59^. In our musical context, *Let-go* performance would make the music less predictable for the audience at certain moments, which could lead to the higher degree of temporal variability in the degree of synchrony. Interestingly, in the context of dyadic interpersonal coordination, it has been suggested that meta-stability, moving in and out of synchrony, is a characteristic of well-functioning interactions^60^. Along this thought, we speculate that the temporal variability of synchrony could signify how much the musical interaction between performers and audience are functioning in an adaptive and flexible manner.

In contrast, decreases of synchrony in the shorter-timescale and increase of diversity in breathing-related pattern by the *Let-go* performance took place more evenly over the whole performances, as shown by the less significant changes in temporal variability. This confirms the idea that the origins of the shorter- and longer-scale sync are different, and further suggests that the shorter-scale sync corresponds to the low-level musical components (shorter beats and meters) and autonomic responses to them, which exist throughout the performances, while the longer-scale sync corresponds to the temporally organized higher-level hierarchical musical structures that are fundamental for the narrative generated by the music performance.

To test our hypothesis 3, we investigated relationship between the audience’s subjective ratings on the performances and the collective movement synchronization. The statistical associations found between changes in psychological ratings and patterns of collective movement suggest that these may be reflecting different manifestations of the same underlying process. Interestingly, results show that higher synchrony in the shorter timescale was negatively associated with the audience’s perception of the innovativeness of performances, which further supports the idea that the shorter-scale synchrony may reflect rather automatic and unconscious alignment to low-level structural/syntactic aspects of the music. That is, the more standard and predictable a performance was (especially in the *Strict* mode), the easier it may have been for the audience to physically and automatically get entrained into it. At the same time, the high predictability may have led to below the optimal zone of uncertainty for music pleasure^61–63^, giving the audience the impression the performance was less innovative. On the contrary, higher synchrony and its temporal variability in the longer timescale was positively associated with the audience’s innovative experience. Thus, the longer-scale synchrony may reflect the audience’s absorption to the dynamics of higher-level musical expression or semantics, which is enriched by the *Let-go* performance mode.

The synchrony scales emerging in the audience can also be interpreted through a musicological and performance analysis perspective. Short timescales are associated with the average duration of beats of reference that create the pulsation of rhythm. The rhythm is more pronounced and metronomic, with more even and rigid beats and less tempo variability in the *Strict* musical performances, enhancing beat-sync. The rhythm of the music has also been previously shown to act as a driver of physiological rhythms such as breathing^37,41^. In contrast, the longer timescales are associated with freer musical gestures, based on deeper, structural pulses in the music, allowing more possibilities in terms of phrasing, articulating and ability to deviate from expectations in *Let-go*^52,64^. The higher music-sync in *Let-go*, as well as the higher temporal music-sync variability, may be caused by the audience’s synchronised response to the spontaneous and unplanned arrival of the ensemble at the same point in the music, crafting moments of peak emotional expression. Previous work has also shown that the audience shows higher physiological synchrony during important structural moments in the music^37^. In future work, we hope to relate both structurally relevant and subjectively intense expressive moments in the music with an analysis of movement synchrony focusing on the time domain.

Here, in terms of musicology, these findings may encourage us to revisit the distinction between the structural design of a composition, and the micro- and macrostructural patterns generated by performers^31,65^. We argue that performers who apply an improvisational state of mind^30^ use a similar kind of generative processes inherent in composition^66^ during the spontaneous creative processes of performance, whether they are performing a repertoire work or freely improvising^64^. Further performance study-based explorations of music performance parameters such as tempo and dynamics in important structural moments in both score and performance, and how they are linked to the subjective experience of musicians and audience, are an important avenue for future work.

The findings in this study have not only scientific significance in and of themselves but also potential applications. Since verbalising and sharing collective creative experiences—exemplified here by a collective music experience—is very difficult, designing and evaluating such experiences is a particularly challenging task. Usually, this endeavor heavily relies on intuitive judgement from domain experts in the target field, such as music directors or concert organisers. The current results involving the objective movement synchrony provide a first step towards the quantification of some aspects of these ephemeral experiences, opening the possibility for sensing technologies to evaluate their elusive yet important aspects. This evaluative information could help the design process by accelerating the try-and-evaluate loop and facilitating knowledge sharing. Furthermore, real-time utilisation of the synchrony information could enhance the experience of all participants immediately. It may be also possible, in some specific artistic contexts, to enrich the audience’s musical experience by converting the synchronisation state into dynamic visual effects or sensations for other modalities and presenting them along with the music.

### Limitations

A major limitation of the current study is the sample size. Although the number of audience members participated in the study is almost double the number of our previous study^30^, the concert was held only once. It will be important to check whether the findings are reproduced in a multiple occasions of concerts with different samples of audience.

The small sample size could have been especially problematic in exploring the effect of audience’s vision, which was investigated through the manipulation of blindfolding on a subset of audience members. In comparison to the within-subject factors such as the performance modes and composition, effects of between-group factors are statistically harder to detect with small samples. In addition, we assigned the blindfolding manipulation based on the seating of the audience members (see Fig. S1). This design choice led us to unbalanced sample size (13 audience members for blindfolded and 29 for non-blindfolded. These problems may have hindered us from detecting potentially significant effect of audience’s vision. Previous studies have shown the role of vision in music appreciation. For example, people depends heavily on visual information when making judgments about music performance^67^. Thus, further experiments with larger sample size would be needed to draw certain conclusion about the relative impact of vision versus audition for the appreciation of the improvisatory performances. In the same way, it is possible that a larger sample size may also reveal interactions between the absorption trait, which is a between-subject variable, and the perception of music with improvised elements.

Other aspects yet to be investigated in the future include the effects of audience members’ personality traits and social relationships between them. For example, a recent study has shown that people’s neural responses to viewing naturalistic stimuli are generally more synchronised with peers who have higher personality similarity^68^. Another study indicated that pairs of strangers, friends, and lovers viewing a series of video clips show different physiological synchrony patterns depending on the emotion elicited by the videos, with a general tendency for higher synchrony between stranger pairs^69^. While the current study did not probe into these aspects, it would be interesting to explore how the composition of audience members might enhance objective synchronisation and subjective musical experience in response to different types of performance approaches.

A technical limitation comes from the way we extracted and measured breathing signals. In physiological synchrony research, special sensors are often used to study breathing rate. In our study, we instead used discrete wavelet transforms and wavelet reconstruction to extract the signals with frequencies associated with breathing rate^70^ from the accelerometer signals. This workflow has been used in previous research, but does indeed require an extra preprocessing step and the signals are not measured directly. Therefore, the breathing results will need to be validated in future studies using a more direct measurement, such as respiration belt, in order to avoid contamination by body movement.

A final limitation comes from the difficulties of organising naturalistic concert-experiments and balancing between a realistic improvisational state of mind experience within the performers, and robust collection of experimental data. The experimental equipment has had effects on the state of flow of the musicians, causing the first performance of Mozart to be more constrained and less innovative, even though the performers were aiming to play in a *Let-go*, improvisational state of mind. This has resulted in a diminshed effect of the mode of performance in the first pair of pieces. Allowing the musicians to get used to the experimental equipment and context will help improve robustness of future experiments.

### Conclusions

In conclusion, this research uncovers the relevance of the often-neglected multiscale coordination between audience and performers, and reveals its deep connections with the quality of the collective subjective experience. Our results provide quantitative evidence that illuminates how a collective music experience is embodied in a multiscale dynamical interaction, which expands the group flow aspects of the relationships between the improvising musicians^19,20^ to a complex dialogue with audiences that is enhanced by the innovative, risk-taking and unexpected qualities of improvisatory performance. Last but not least, the reported results highlight the importance of regarding collective creative activities as physically embodied experiences, suggesting that a rich tapestry of physical behaviour is underlying the shared experience even in audiences that are often regarded as passive.

## Methods

### Experimental procedure

The concert/experiment involved the Portorius String Quartet, who performed movements from Mozart (String Quartet No. 15 in D Minor K. 421 - first movement: Allegro moderato) and Haydn (String Quartet in G Major, Hob.III:75, Op. 76, No. 1—third movement: Menuetto: Presto) as well as improvised pieces in different performance modes (Table S1). Specifically, for the repertoire works, the same piece was performed twice, in each of the two modes, *Strict* and *Let-go*, varying the order, allowing us to better isolate the effect of performance mode on the audience.

The two repertoire pieces were chosen as they are both from the classical period and their phrase structure lends itself to more straightforward creative work when performed in *Let-go*, but they contrast each other in mood and musical energy. Mozart’s piece is more introverted and complex from a contrapuntal point of view. Haydn’s piece is more extroverted and varied from a rhythmic point of view. The four improvisation pieces (pieces 3–6 in Table S1 were fully improvised and very different between each other, making it difficult to compare in terms of performance modes. Therefore, they were excluded and only the four repertoire pieces were analysed in this study. Audio-video recordings of the four repertoire pieces are shared on the Open Science Framework server (https://osf.io/ar64j/).

Prior to the concert, all members of the quartet have taken part in Professor David Dolan’s course *Interpretation through Improvisation* at the Guildhall School of Music and Drama in London^71^. The method taught during the course involves a creative approach to studying and performing repertoire works, engaging with structural, harmonic, rhythmic and motivic reductions while maintaining an improvisational state of mind, encouraging risk-taking and allowing spontaneous deviations from the score in coordination with ensemble partners. As such, the string quartet was able to adopt the different states of mind and approaches required to perform under the two different modes.

The concert experiment was conducted in a recital room in the Guildhall School of Music and Drama (see Fig. S1).

### Participants

Audience members were recruited via posters on bulletin boards and online call for participation. Fifty adult volunteers attended the concert experiment as audience. They were mainly graduate students and staff of the Imperial College London or their families and friends, with a wide range of experience with classical music. Out of them, 8 subjects encountered issues with the physical motion recording or failed in giving the subjective ratings on the performances. Therefore, the data from the remaining 42 subjects were subjected to the analyses. Here we must note a diversity of nationalities, in particular 12 different nationalities from Europe, 4 different nationalities from Asia, plus 2 Australians and 1 Mexican. Moreover, unlike the usual distribution of classical music attendees, most of our participants were under 30, and also female. A large proportion were familiar with playing music, but only a subset were formally trained or actively practising. Further details on the characteristics of the audience members are provided in the supplementary materials (Section A.1). In order to investigate the role of audience’s vision, 13 out of the 42 audience members listened to the performances wearing blindfolds.

### Measurements

#### Body motion acceleration

The performers’ head motions were measured with inertial measurement units (IMUs; TSND151; ATR-Promotions, Japan) placed on the middle of their forehead, attached to the fNIRS brain activity measurement device (HOT-1000; NeU, Japan). The audience members’ body motion fluctuations were measured with IMUs contained in the smartphones (Zenfone 3 Laser; ASUSTek, Taiwan) that they wore around their necks^72^. The sampling frequency was 100Hz for both sensors, and then downsampled to 50 Hz.

#### Questionnaires

Before the study, audience members filled a psychometric questionnaire to assess their psychological trait of absorption^73^ as this has been previously related to the enjoyment of music^44^, as well as susceptibility to altered states of mind and even psychedelic experiences^45^.

After each pair of successive performances, the audience members rated their subjective evaluation of each performance on five items: how they felt each performance to be (1) Improvisatory, (2) Innovative, (3) Emotionally Engaging, (4) Musically Convincing, and (5) Risk-taking. These items were identical to the ones used in the previous studies^29,30^. Two additional items were added, where the audience members were asked to rate their degree of (6) familiarity with the piece and (7) sleepiness. The rating for each of these seven items was given on a six-level Likert scale, ranging from 0: “not at all/none” to 5: “totally/completely”. In the questionnaire, the pair of performances were labeled as “performance 1” for the earlier one and “performance 2” for the latter one, and the items are presented for each of them.

The collected rating data contained small amount of missing values; in the total number of 1176 values consisting of 168 observations (42 members $\times$ 2 pieces $\times$ 2 repetitions with different modes) and the seven questionnaire items, “Improvisatory”, “Convincing”, “Familiar”, and “Sleepy” items had one missing value each, “Risk-taking” item had two missing values. For each subject and each performance there was no more than one missing value. These missing values were imputed using the missForest algorithm, a random forest-based multiple imputation scheme^74^, as the PCA on the questionnaire scores requires complete dataset.

### Analysis

#### Wavelet synchrony analysis

To evaluate synchrony between physical activity, triaxial head acceleration data of the musicians (from IMU sensors) and body acceleration data of the audience (from smartphones) was converted to a one-dimensional time series of Euclidean norm of acceleration.

$$\begin{aligned} a(t) = \sqrt{a^2_x(t)+a^2_y(t)+a^2_z(t)} \end{aligned}$$

Then, we evaluated movement synchrony of each pair of signals by using the WTC^46^ of their acceleration norm time series. WTC finds regions in time-frequency space where two time series covary, but do not necessarily have high power. WTC has been used to evaluate interpersonal movement synchrony in various types of interactions^18,48,50^ and is defined as^75^:

$$\begin{aligned} R^2(t, s) = \frac{\vert S (s^{-1}W^X(t, s) W^Y(t, s)) \vert ^2}{S ( s^{-1} \vert W^X(t, s)\vert ^2) S (s^{-1} \vert W^Y(t, s)\vert ^2)} \end{aligned}$$

where $W^X$ and $W^Y$ refer to the wavelet transforms of the two signals and *t* and *s* refer to time sample and wavelet scale. Wavelet scale *s* is directly associated with a Fourier period^75^, which is used to discuss scales of synchrony. Results were computed using the open-source wavelet-coherence Matlab package^76^, with it’s default setting of the Morlet wavelet $\psi _0(\eta ) = \pi ^{-\frac{1}{4}}e^{i\omega _0 \eta }e^{-\frac{1}{2}\eta ^2}$ with the parameter $\omega _0 = 6$ as the wavelet function, and the scale resolution of 12 scales per octave.

To calculate the synchrony between performers and audience members (P-A sync) for each performance, first, for each performer *X* and audience member *Y*, the WTC coefficient $R^2(t, s)$ was time-averaged over the duration of the performance ($\langle R^2(s) \rangle$). Then, for each audience member *Y*, the coefficients were averaged over all the four performers (*X*s), resulting in a measure of how much each listener was in sync with the performers on average, at each timescale *s*, for each performance.

Similarly, to quantify the synchrony between audience members (A-A sync), firstly we temporally averaged the WTC coefficient $R^2(t, s)$ over the duration of the performance ($\langle R^2(s) \rangle$) for all the audience pairs (*X*, *Y*). Then, for each audience member *X*, the coefficients with all other audience members (*Y*s) were averaged. This provides a measure of how much each audience member was in sync with other audience on average, at each timescale, for each performance.

Due to the similar duration of the repertoire pieces (between 120 and 140 seconds), the same wavelet scales (or Fourier period) can be used to discuss all pieces. We choose a range of relevant periods to be ${<}$ 0.5 s, as audience’s physical activities in the timescales below it would have no musically meaningful counterpart.

The synchrony analysis is conducted in order to identify ranges of frequencies (or bands) where there are significant differences in the audience’s degree of synchrony between the performance modes. Averaging the per-subject wavelet coefficients in these bands provides a measure of synchrony in that band, which can be used further to test how the synchrony in these different bands are correlated with the audience’s subjective perception (hypothesis 3), as well as how the effect of the performance modes on the synchrony is affected by other factors such as audience’s sight (blindfolding) and compositions of the pieces (see the explanation on the multilevel model below).

#### Breathing rate analysis

To further investigate A–A sync, the breathing rate of participants was extracted from the front (z-axis) of the triaxial acceleration data by using a continuous wavelet transform^70^. The wavelet coefficients in the relevant scales for breathing (3–5 s) were then used to reconstruct the respiration signals^53^, producing a time series that can be analysed with stationary methods, due to the oscillatory nature of breathing. Due to the nature of this extraction procedure, we note that there is a possibility that a part of the breathing rate signals might have resulted from body-swaying to the music.

To investigate synchrony of breathing, average pairwise PLV^56^ was computed and averaged for each subject. PLV is a measure of phase synchrony between a pair oscillatory signals calculated using their average phase difference. To obtain mean synchrony for a subject, their mean PLV with all other audience members is computed.

To investigate variability in breathing, ER was computed on each listener’s reconstructed breathing signals using the state space estimator^54^. This measure uses vector auto-regressive model to estimate ER of continuous signals and is shown to be data-efficient and calibrated against other measures like Lempel-Ziv complexity (LZc).

#### Music performance analysis

To quantitatively study the music, a number of techniques for the analysis of performance^77^ are used on the audio data, focusing, in particular, on tempo and dynamics.

To understand the timescales of movement synchrony emerging between the performers and the audience, it is important to establish a performance-related *beat of reference*, which is the conducting beat to which the performers relate. This beat of musical or expressive reference is related to the tempo of the performance as well as to the time signature, and functions as the smallest unit of musical meaning for the given performance. Note that Haydn’s 3^rd^ movement from *Op. 76 no. 1* performed in this concert (marked *presto* and written in 3/4) is much faster than Mozart’s 1^st^ movement from *K. 421* (marked *Allegro moderato* and written in 4/4). In Haydn’s piece, the performers related to one whole bar as a basic beat of musical reference, whereas in Mozart’s piece, half a bar was the essential beat of musical reference. At times, the musicians created one uninterrupted gesture made of two or four bars in the case of Haydn’s movement and one or two bars in the case of Mozart. This has been confirmed through critical listening sessions conducted independently by each quartet member, and David Dolan, a co-author of this article. This is why, when comparing mean and variability between modes of performance, we relate to beats of musical reference and gestures rather than metronomic pulses of crochets or quavers.

Analysis of the tempo requires quantitatively measuring the duration of each bar. This was done by making use of open-source software Sonic Visualiser^57^. From this data, the mean and standard deviation of each bar’s duration were computed. These durations were interpreted with respect to the time signature of the piece.

To analyse the dynamics of the performance, first the dual-channel audio was converted to mono, by averaging the two channels, then the loudness curve was computed in loudness units (LUFS)^78^ using the mzPowerCurve plugin in Sonic Visualiser^57^ at a sample rate of 200, with a moving window of 480 samples.

As this study makes use of complex, often 2 minutes long pieces of music, some techniques for analysis of timbre, such as pitch, are difficult to interpret here. On the other hand, we made use of entropy as a measure of music complexity^79^. Entropy of the loudness curve, and spectral entropy, respectively, were computed using lziv_complexity and spectral_entropy in package antropy^80^ the former on the loudness curve, the latter both on the loudness curve and directly on the mono WAV audio data.

### Statistical tests

To study the differences in ratings, as well as in the average synchrony and temporal variability of synchrony at each period (timescale) between performances, we estimated a three-way mixed-effect multilevel model that includes the performance mode and composition as within-subject factors and blindfolding as a between-group factor with fixed effects, and each subject as random effects. We primarily focused on the main effect of the performance mode, as it directly addresses our hypotheses 1 and 2. The main effect of blindfolding and its interaction with performance mode address the impact of visual information available to audience. The composition was included as a controlling variable, so its effects were of little interest at the stage of experimental design. Using the lme4 package^81^ in R statistical software, the multilevel model is expressed as

$$\texttt{DV}\, {\sim}\,\texttt{Blindfold\,*\,Composition \,*\, Mode + (1|Subject)} \\ \ + \texttt{(1|Composition:Subject)}+\texttt{(1|Mode:Subject)}$$

where DV represents the dependent (target) variable.

When DV is a subjective rating for each performance from audience members, the main effect of Mode in the model addresses hypothesis 1. When DV is synchrony, its temporal variability, or a measure derived from the breathing-related patterns, the main effect of Mode addresses hypothesis 2. All the fixed-effect independent variables are zero-centered before estimation^82^. Statistical significance of the variables were tested using the lmerTest package^83^. To quantify the fitting of the entire model, marginal and conditional $R^2$ values are calculated using MuMIn package^84^. Diagnostic analysis of the multilevel models was conducted using DHARMa package^85^. By checking the distribution of residuals, its variance over the predictor variables or fitted values, we have confirmed the normality and homoscedasticity, with no conspicuous deviations or outliers.

In the analyses of movement synchrony, to correct for multiple testing over many timescales, false discovery rate (FDR) control via the Benjamini-Hochberg procedure^86^ was applied to the *p*-values.

For each subject and performance, the mean movement synchrony values in the timescales with significant *Let-go* < *Strict* difference were averaged into the beat-sync measure, those with significant *Let-go* > *Strict* difference were averaged into the music-sync measure. Similarly, synchrony variability values in the timescales with significant *Let-go* > *Strict* were averaged into the music-sync variability measure.

To investigate how these measures were predictive of the subjective evaluations by the audience (hypothesis 3), multilevel models of the form rating $\sim$ sync + (1|Subject) were tested. Here, sync represents either the beat-sync, music-sync, or music-sync variability after centering-within-cluster. This analysis is equivalent to the within-subject repeated measures correlations^87^, and evaluates how the within-subject variances in the sync and rating are consistently correlated over the four performances (*Let-go* and *Strict* performance mode for the both pieces) or over the two performances with the different modes for each piece, separately.

Multilevel models of the form rating $\sim$ Absorption *   Mode + (1|subject) are used to quantify the effect of absorption on performance ratings and its interaction with the performance mode.

## Supplementary Information

Supplementary Information.

## Data Availability

Data generated as part of this study are available to other investigators exclusively for the purposes of basic research exploring the relationship between ensemble music experience and physical as well as brain activities. Data is available from the corresponding author on reasonable request.
