# Supplementary material for: Multiscale synchronisation dynamics reveals the impact of an improvisatory approach to performance on music experience
Source: Sci Rep. 2025 Mar 24;15:10097. doi: 10.1038/s41598-025-90271-1 (PMC11933434; doi:10.1038/s41598-025-90271-1)
Supplement: Supplementary file 1 — Supplementary Information. [file 41598_2025_90271_MOESM1_ESM.pdf]

Supplementary Material: Multiscale Synchronisation Dynamics Reveals the Impact of an Improvisatory Approach to Performance on Music Experience

Takayuki Nozawa*[1,2], Madalina I. Sas*[3], David Dolan[4], Hardik Rajpal[3], Fernando E. Rosas[3,5], Christopher Timmermann[3], Pedro A.M. Mediano*[3], Keigo Honda[1], Shunnichi Amano[1], Yoshihiro Miyake[1], and Henrik J. Jensen[3,1]

[1]Tokyo Institute of Technology, Tokyo, Japan
[2]University of Toyama, Toyama, Japan
[3]Imperial College London, London, UK
[4]Guildhall School of Music and Drama, London, UK
[5]University of Sussex, Brighton, UK

A Experimental setup

The programme of performances is shown in Table S1. The seating layout for the performers and audience members is shown in Fig. S1.

Piece	Type	Composer	Performance mode	Blindfolding
1	repertoire	Mozart	*Let-go*	yes
2	repertoire	Mozart	*Strict*	yes
3	improvisation	—	*Strict*	no
4	improvisation	—	*Strict*	no
5	improvisation	—	*Let-go*	no
6	improvisation	—	*Let-go*	no
7	repertoire	Haydn	*Strict*	yes
8	repertoire	Haydn	*Let-go*	yes

Table S1. Performance programme. Note that only the four repertoire pieces 1, 2, 7, 8 were analysed in the current study.

A.1 Detailed characteristics of audience members

At the recruitment stage, we asked listeners to fill in a form for their demographic information, including their gender, age range, nationality, musical experience (both playing and listening), and preference for music genres. Out of the 42 audience members whose data are analysed in this study, 35 members voluntarily filled in the form.

Demographics

Out of the 35 listeners, 22 were female and 13 were male. Regarding their age ranges in years, two were in 16–20, 17 in 21–25, eight in 26–30, three in 31–35, two in 36–40, and one each in 56–60, 61–65, and 66–70, respectively. As for their nationalities, most were from Europe (10 British; 3 Romanian; 2 French, and 2 Spanish; 1 Danish, Finnish, Irish, Italian, Portuguese, Russian, Slovak, Swiss), followed by Asia (2 Chinese, 2 Indonesian, 1 Indian, 1 Israeli), Oceania (2 Australian) and Latin America (1 Mexican).

Experience with playing music

We asked four questions about their experience with playing music:

- *”For how many years have you played a musical instrument (including voice)?”* with 11 options: 0, 1, 2 .. 9, 10+ years. Twenty-five out of the 35 respondents selected ”10 or more years”; four selected ”0 years”; two selected ”5 years”; and one each selected ”1 year,” ”3 years,” ”6 years,” and ”8 years,” respectively.

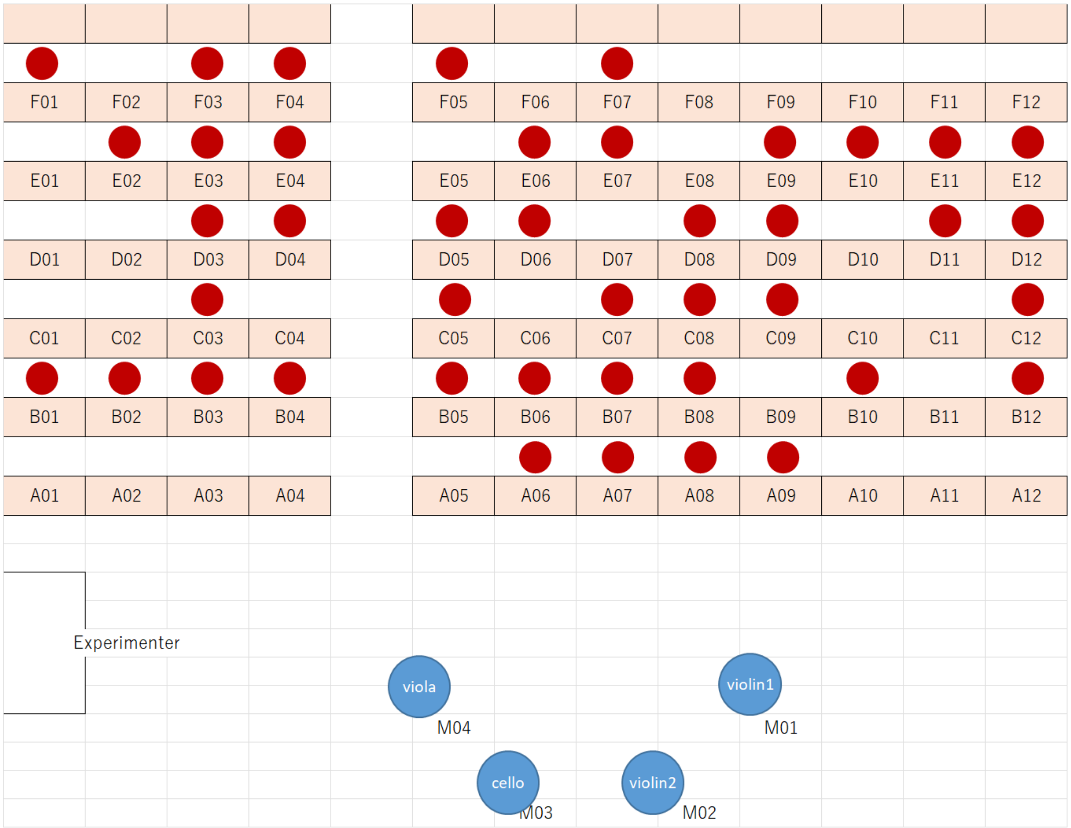

Figure S1. Recital room layout for the concert experiment. Blue circles represent performers, and the red circles represent audience members. Thirteen audience members in the seats numbered 01 to 04 wore blindfolds during the repertoire pieces.

- *"How many years of formal training on a musical instrument (including voice) do you have?"* with 7 options: 0, 1, 2 .. 5, 6+ years. Twenty respondents selected "6 or more years"; five selected "0 years"; four selected "5 years"; and two each selected "1 year," "2 years," and "3 years," respectively.
- *"How many years of training in formal music theory have do you have?"* with 7 options: 0, 1, 2 .. 5, 6+ years. Fourteen respondents selected "6 or more years"; seven selected "0 years"; five selected "3 years"; four selected "1 year"; two each selected "4 years" and "5 years," respectively, and one selected "2 years."
- *"For how long do/did you practice for in a day?"* with 7 options: 0, 1, 2 .. 5, 6+ hours. Fifteen respondents selected "0 hours"; seven selected "1 hour"; five selected "3 hours"; four selected "4 hours"; three selected "2 hours"; and one selected "5 hours."

Summarising these responses, the majority of our responding participants (31/35) have played a musical instrument, with the majority (25/31) having played an instrument for more than 10 years. Even so, almost a half (15/35) do not, or no longer, practice an instrument daily.

Engagement in music-listening

We also asked questions about how they are engaged in listening to music, and what genres they prefer, as well as their familiarity with classical music.

- *"How many musical events did you attend in the last year?"* with 7 options: 0, 1, 2 .. 5, 6+. Twenty-five respondents selected "6 or more events"; four selected "3 events"; two selected "4 events"; and one each selected "0 events," "1 event," "2 events," and "5 events," respectively.
- *"How much time do you spend attentively listening to music in a day?"* with 6 options: 0, 1, 2, 3, 4, 5+ hours. Fourteen respondents selected "2 hours"; ten selected "1 hour"; four selected "5 or more hours"; three selected "3 hours"; two selected "0 hours"; and one selected "4 hours". One did not provide the answer.

- *”What musical genres do you like?”* with 5 options: “Rock”, “Classical”, “Jazz”, “Electronic”, “Other” with a fill-in field, asking respondents to check all that apply. Thirty respondents selected ”Classical”; twenty-seven selected ”Jazz”; twenty-three selected ”Electronic.” ”Other” included ”pop,” ”folk,” ”blues,” ”choir,” ”hip-hop,” ”indie,” ”world,” and so on.

- *”Please rate your familiarity with classical and early romantic European art-music (including composers like Haydn, Beethoven and Schubert)?”*, with 6 options. No respondent selected ”0: Never heard the composer’s music”; two selected ”1: Occasionally heard the composer’s music”; nine selected 2: I sometimes listen to the composer’s music; ten selected ”3: I regularly listen to the composer’s music”; nine selected ”4: I have studied aspects of the composer’s music”; and five selected ”5: I have studied this composer’s music in detail.”

B Psychology and performance ratings

B.1 Combined effects of mode, composition, and blindfolding on performance ratings

Results for the effects of composition and visibility are shown in Fig.S2 and Table S2. They revealed significant interaction between the performance mode and composition factors for the Improvisatory, Innovative, Risk Taking, Familiar ratings and PC1, main effects of performance mode for the Improvisatory, Innovative, Risk-taking, Engaging ratings and PC1, and main effects of composition for the Improvisatory and Innovative ratings and PC1.

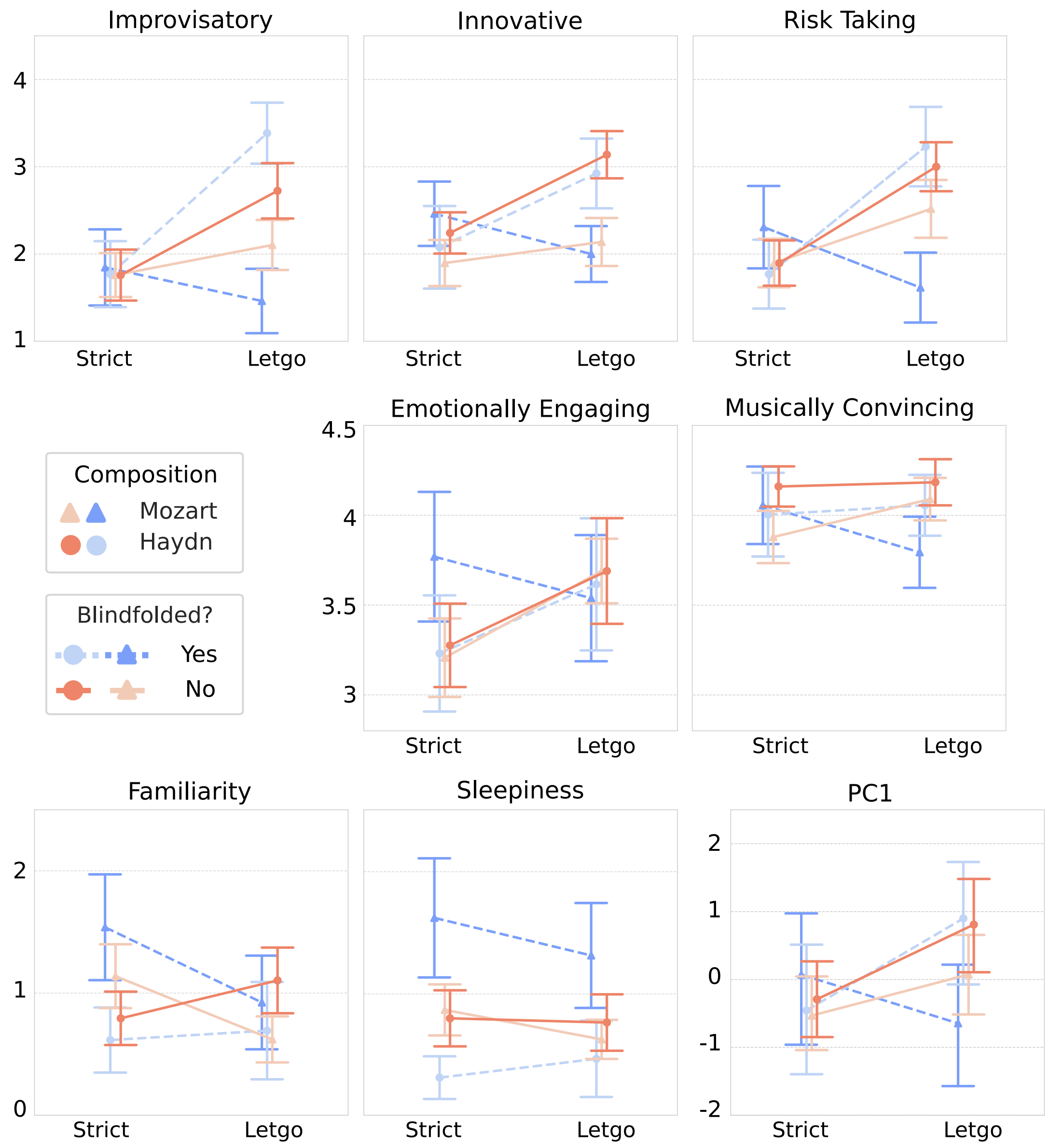

Figure S2. Comparing the audience's perception of modes of performance, separately in each of the two repertoire pieces (by Mozart and Haydn), and grouping by whether the audience was blindfolded. Error bars show standard error of mean (SEM). The audience perceives the Haydn *Let-go* performance as much more Improvisatory, Innovative, and Risk-taking than the *Strict*, while there is little difference in these two metrics in the Mozart piece, echoing the performers' reports on the success of the performance.

Visibility (sighted vs. blindfolded) had no significant main effects nor interaction with performance modes on the ratings. Although not significant, blindfolded audience members tended to show less sensitivity to the performance modes than sighted ones. There were also significant 3-way interactions between visibility, performance mode and compositions for some rating items.

Effects of	statsistics	Improvisatory	Innovative	RiskTaking	Engaging	Convincing	Familiar	Sleepy	PC1
Main effects									
Sight	β	0.01	0.00	−0.03	0.03	−0.07	−0.01	0.06	−0.01
	t_{40}	0.08	0.03	−0.25	0.25	−0.65	0.08	0.57	−0.11
	p-value	0.940	0.974	0.801	0.800	0.522	0.934	0.575	0.910
Composition	β	**−0.32**	**−0.38**	−0.21	0.04	−0.23	0.10	0.26	**−0.28**
	t_{40}	**−2.54**	**−2.86**	−1.63	0.22	-1.56	0.73	1.56	**−2.10**
	p-value	**0.015**	**0.007**	0.110	0.823	0.126	0.469	0.127	**0.042**
Mode	β	**0.41**	**0.31**	**0.44**	**0.27**	0.07	−0.12	−0.10	**0.39**
	t_{40}	**3.78**	**2.69**	**3.75**	**2.44**	0.51	−1.30	−1.07	**3.12**
	p-value	**<0.001**	**0.011**	**< 0.001**	**0.017**	0.615	0.198	0.292	**0.003**
2-way interactions									
Sight × Composition	β	−0.18	0.13	−0.09	0.10	0.06	0.23	0.42	−0.01
	t_{40}	−1.44	0.97	−0.67	0.58	0.37	1.67	**2.58**	−0.04
	p-value	0.158	0.336	0.505	0.566	0.713	0.103	**0.014**	0.967
Sight × Mode	β	−0.01	−0.12	−0.14	−0.14	−0.15	−0.06	0.02	−0.14
	t_{40}	−0.11	−1.03	−1.16	−1.26	−1.07	−0.64	0.25	−1.12
	p-value	0.914	0.307	0.254	0.213	0.291	0.522	0.802	0.269
Composition × Mode	β	**−0.67**	**−0.59**	**−0.62**	−0.11	0.05	**−0.60**	−0.24	**−0.56**
	t_{40}	**−3.67**	**−3.60**	**−2.97**	−0.52	0.21	**−3.30**	−1.33	**−3.20**
	p-value	**<0.001**	**<0.001**	**0.005**	0.603	0.833	**0.001**	0.192	**0.003**
3-way interaction									
Sight × Composition × Mode	β	**−0.41**	−0.21	**−0.48**	−0.25	−0.33	0.05	−0.10	**−0.42**
	t_{40}	**−2.23**	−1.27	**−2.29**	−1.16	−1.52	0.26	−0.55	**−2.37**
	p-value	**0.031**	0.213	**0.027**	0.251	0.138	0.794	0.588	**0.023**
Fitting of the entire model									
	Marginal R^2	0.111	0.089	0.102	0.029	0.031	0.042	0.069	0.091
	Conditional R^2	0.663	0.729	0.557	0.523	0.514	0.661	0.682	0.690

Table S2. Combined statistical effects on the audience ratings identified using the multilevel model. In the multilevel model, for the binary variables Sight, Composition, and Mode, we used visible, Haydn, and *Strict* as references, and estimated the effects of blindfolded, Mozart, and *Let-go*. β represents the standardized coefficient.

Composition-wise, the blindfolded audience tended to feel more improvisatory and risk-taking toward the *Let-go* mode performance of the Haydn's composition, but they felt oppositely to the Mozart's composition. We surmise that visual perception could affect the music listening experience to some extent, while the limited sample size and the unbalanced manipulation of blindfolding may have prevented us from detecting clear effects.

B.2 Effect of absorption on ratings

We collected responses to the absorption questionnaire[1] from the 42 audience members (mean = 78.65, SD = 22.04). The lowest and highest absorption scores in the audience were 23, and 121, respectively. For the purpose of better understanding the relationship between psychological traits in the audience and their experience at the concert, we first looked for statistical links between absorption and the performance ratings.

Results of linear modelling shows absorption is significantly linked to the first principal component ($\beta = 0.27, t_{39} = 2.64$, $p = 0.012$), as well as to the Innovative ($\beta = 0.25, t_{39} = 2.30, p = 0.027$), Familiar ($\beta = 0.27, t_{39} = 2.38, p = 0.022$), and most significantly, Emotionally Engaging ratings ($\beta = 0.30, t_{39} = 3.14, p = 0.003$). There are no significant main effects of mode of performance or interactions between absoprtion and mode of performance. Complete results of the linear modelling for all variables are in Table S3.

Rating	β	t_{39}	p-value	
PC1	0.27	2.64	0.012	*
Improvisatory	0.20	1.79	0.080	
Innovative	0.25	2.30	0.027	*
RiskTaking	0.20	1.91	0.064	
EmotionallyEngaging	0.30	3.14	0.003	**
MusicallyConvincing	−0.01	−0.07	0.947	
Familiar	0.27	2.38	0.022	*
Sleepy	−0.03	−0.13	0.894	

Table S3. Linear modelling results for the relationship between individual absorption metric and the listeners' performance ratings. β represents the standardized coefficient for the effect of absorption on each rating.

C Physiology

C.1 Audience's physical motion recording data

Before the experiment, we have confirmed that the IMU sensors of the smartphones are appropriately working, by checking applied reorienting and shaking are correctly recorded in their log files. We have also confirmed that the recorded data show no floor and ceiling effects, by visual inspection of time series plot and histogram for each recording.

We did not conduct calibration for precise baseline. As a result, there were differences in the baseline values between the sensors, as indicated by the distribution of the raw values of acceleration norms combined for all subjects (Fig. S3(a)). On the other hand, when the acceleration norms were mean-centered for each subjects to account for the differences in the baseline values between the sensors, the combined distribution converges to a unimodal distribution, as shown in (Fig. S3(b). Note that the WTC and other analysis methods used in the study focus on the relative temporal variation and co-variation of the signals, thus are insusceptible to the differences in the baseline.

C.2 Effect of composition

The performers reported that they failed to achieve the ideal *Let-go* performance in the first performance of Mozart (Piece 1). Audience members' perception was in accordance with this judgment by the performers. Therefore, to incorporate this difference in the performance quality of the *Let-go* mode, we conducted the synchrony analyses separately for the two performances of each repertoire piece.

C.2.1 Audience's movement

To evaluate power of physical activity in different periods, a wavelet power spectrum (WPS), given by $\|W^X(t,s)\|^2$, was applied to the acceleration norms. Log-scaled WPS was averaged over the time duration and subjected to group-level ANOVAs at each period. Bias in the wavelet power spectrum (WPS) was rectified using the method of[2].

Fig. S4 shows mean power spectra of the audience's movement during the two performances of the two repertoire pieces.

The power spectra indicate the existence of oscillatory components at the periods around 1.5s and 3s, possibly reflecting the audience's physiological signals (heartbeat and respiration) or their implicit bodily reaction to musical beats. We explore this

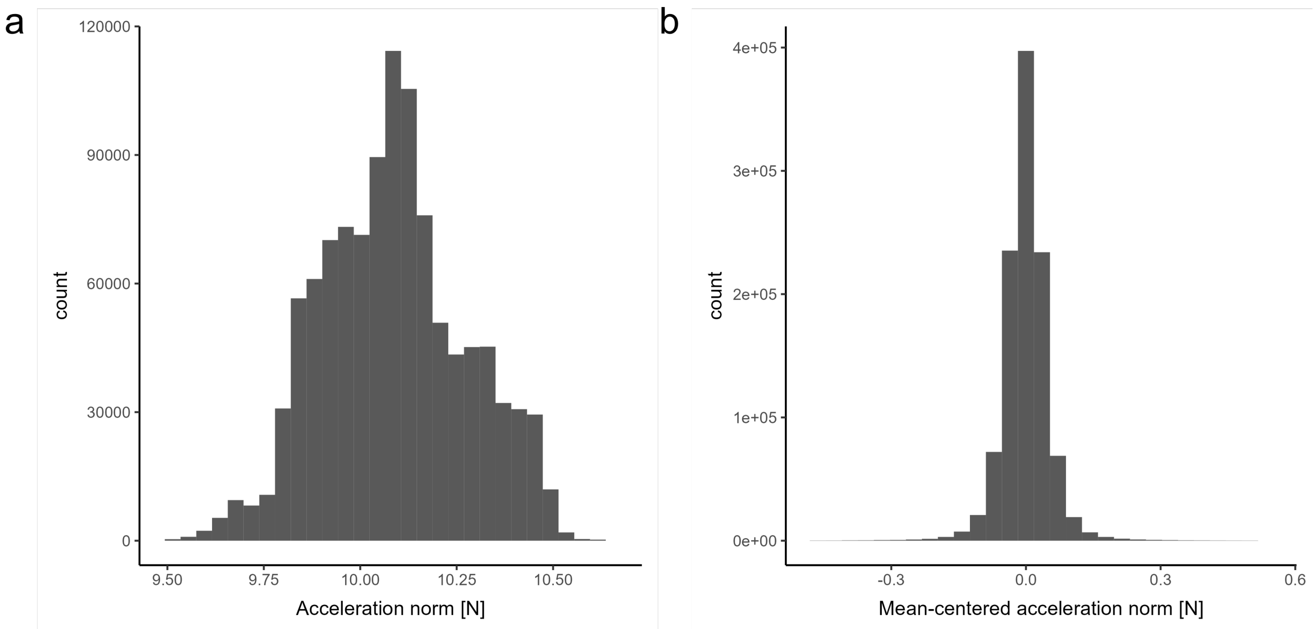

Figure S3. Distribution of the acceleration norms computed from the triaxial physical motion data of the audience. (a) Raw acceleration norm of all the audience members are combined. (b) To account for the difference in the baseline values between sensors, mean value was subtracted from the acceleration norm for each subject and then combined over subjects.

relationship further in the sections that follow 2.2. Comparing the two performance modes, whilst no significant differences were found, we can observe trends by analysing the two pairs of performances separately. The audience showed a tendency towards larger amplitude movement during the *Let-go* performance compared to the *Strict* performance of Haydn's piece. The tendency was less clear between the *Let-go* and *Strict* performances of Mozart's piece.

C.2.2 Movement synchrony

When studying the synchrony regimes individually for each piece (Figs. S5 and S6), we observe the peaks and troughs in the time-averaged synchrony differ according to the piece being performed, thus explaining the interactions between performance mode and composition at certain periods.

C.2.3 Temporal variability of movement synchrony

Temporal variability of synchrony was higher in the *Let-go* mode in longer timescales for both compositions (Figs. S7 and S8). Note that the temporal variability is commonly lower in longer timescales because of the higher auto-correlation of synchrony (i.e. the longer the timescale is, the slower the synchrony changes, limiting variability) and the more limited available time range due to the exclusion of the cone of influence to avoid edge effects in the WTC analysis.

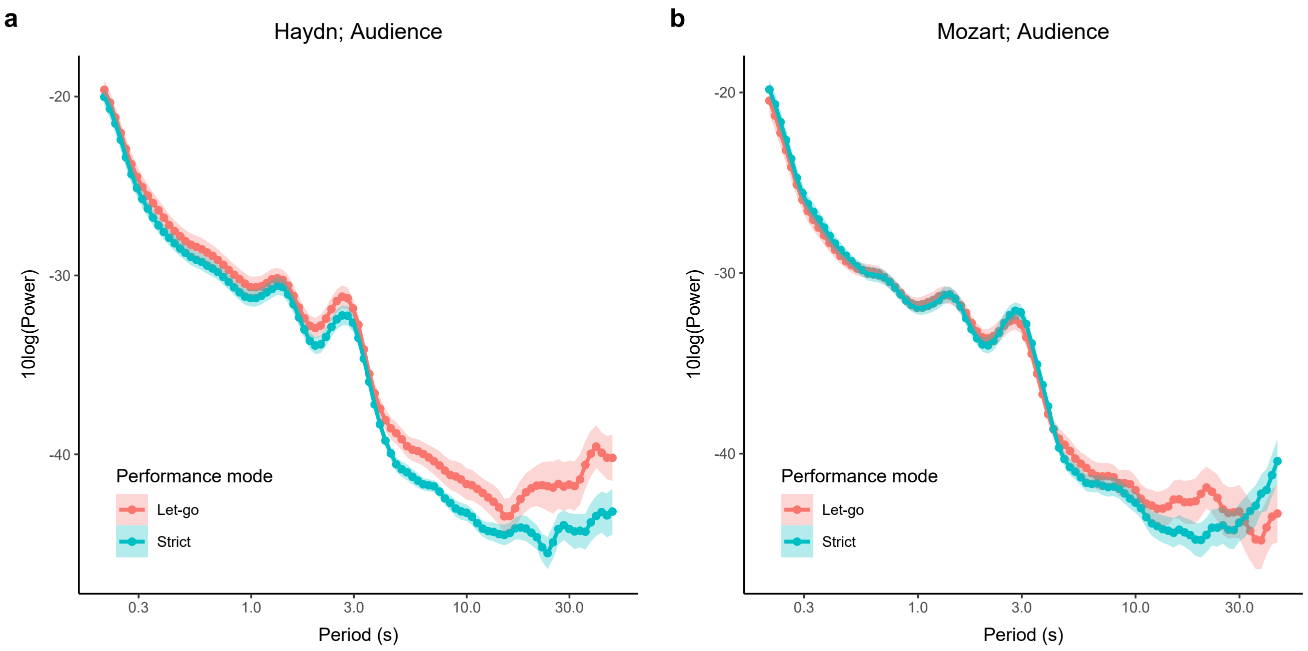

Figure S4. Mean power spectra of the audience's physical activity during the two performances of (a) Haydn's piece and (b) Mozart's piece, both in the *Let-go* and *Strict* performance modes. Spectra are calculated by time-averaging the log-transformed wavelet power in each performance. For the Haydn pieces, the effect is more pronounced, showing higher power during the let-go performance, yet the effects are not significant.

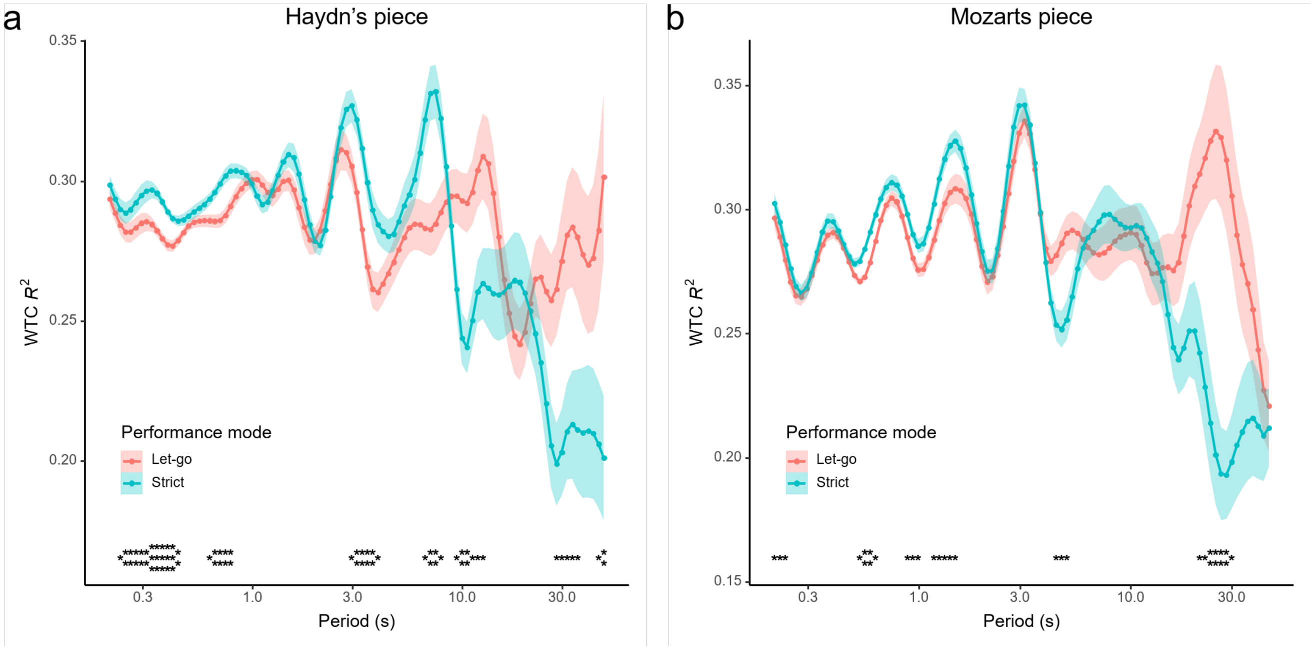

Figure S5. (a) Mean P-A sync over different timescales (periods) for the *Let-go* and *Strict* performances of Haydn's piece. (b) Mean P-A sync over different timescales for the two performances of Mozart's piece. Shaded areas represent SEM over 42 subjects. Periods with significant differences are marked by asterisks. *: $p < 0.05$; **: $p < 0.01$; ***: $p < 0.001$; FDR-corrected.

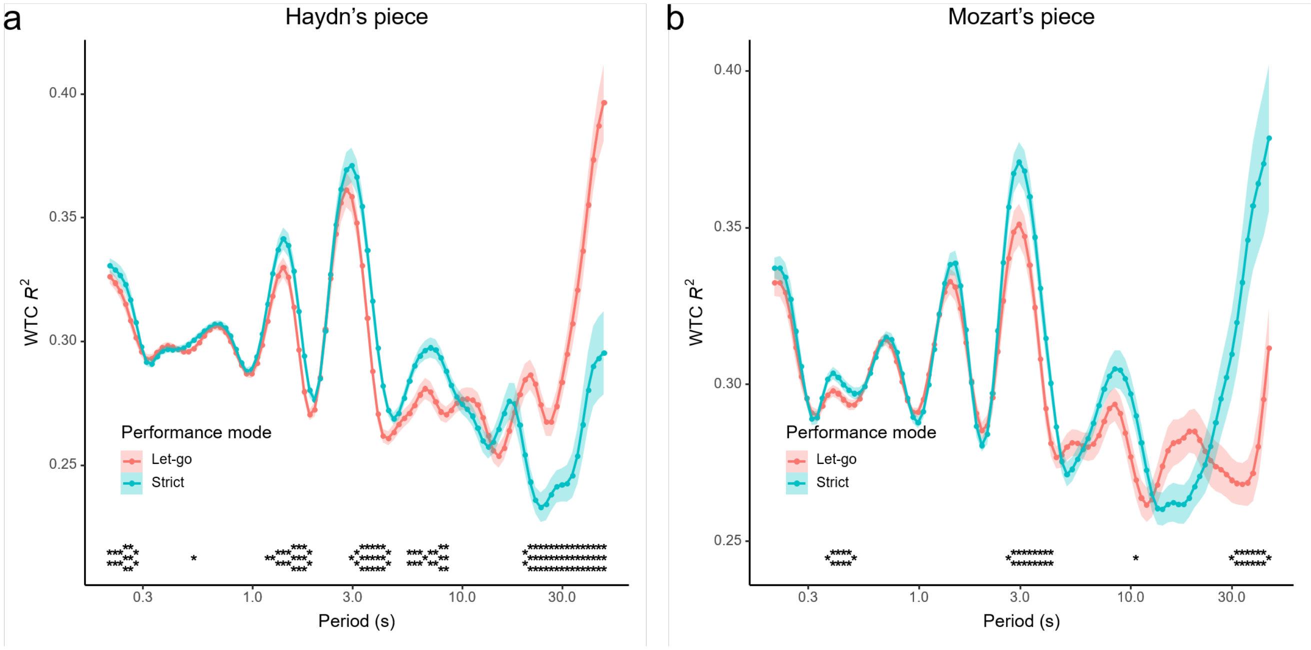

Figure S6. (a) Mean A-A sync over different timescales (periods) for the *Let-go* and *Strict* performances of Haydn's piece. (b) Mean A-A sync over different timescales (periods) for the two performances of Mozart's piece. Shaded areas represent SEM over 42 subjects. Periods with significant differences are marked by asterisks. *: $p < 0.05$; **: $p < 0.01$; ***: $p < 0.001$; FDR-corrected.

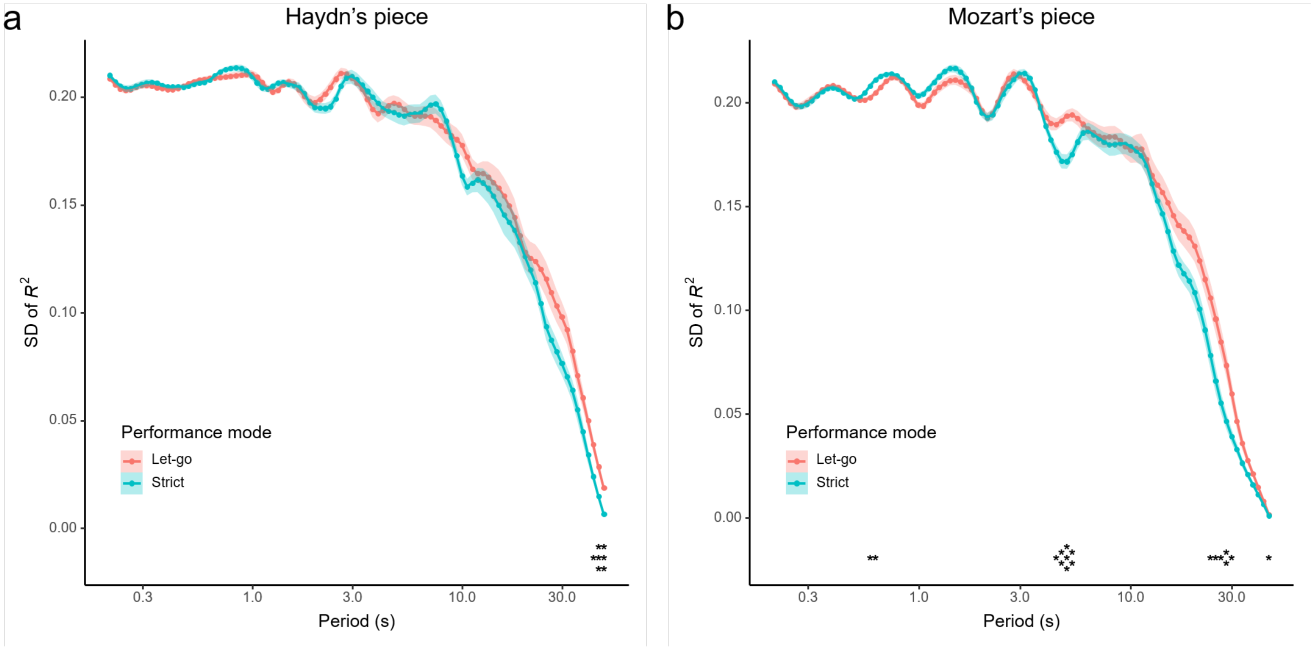

Figure S7. (a) Temporal variability (standard deviation over time) of the P-A movement sync at different timescales (periods) for the *Let-go* and *Strict* performances of Haydn's piece. (b) Mean temporal variability of the P-A sync at different timescales for the two performances of Mozart's piece. Shaded areas represent SEM over 42 subjects. Periods with significant differences are marked by asterisks. *: $p < 0.05$; **: $p < 0.01$; ***: $p < 0.001$; FDR-corrected.

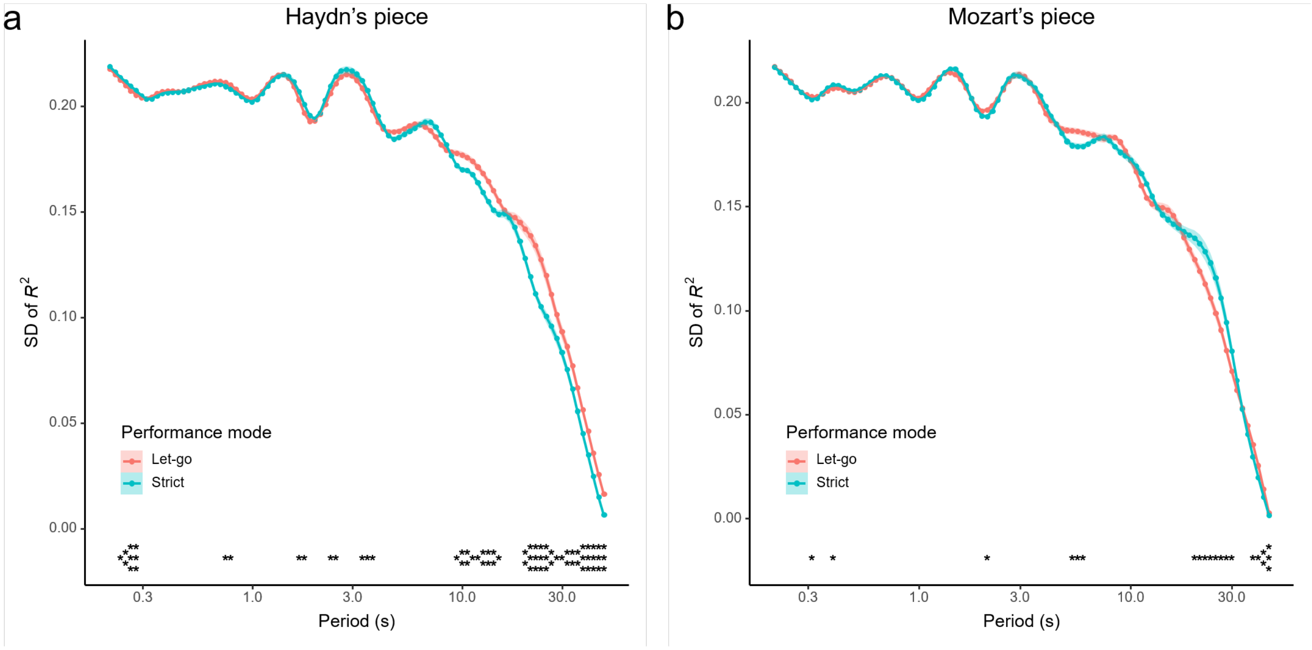

Figure S8. (a) Temporal variability (standard deviation over time) of A-A sync at different timescales (periods) for the *Let-go* and *Strict* performances of Haydn's piece. (b) Mean temporal variability (standard deviation over time) of A-A sync at different timescales for the two performances of Mozart's piece. Shaded areas represent SEM over 42 subjects. Periods with significant differences are marked by asterisks. *: $p < 0.05$; **: $p < 0.01$; ***: $p < 0.001$; FDR-corrected.

C.3 Higher order correlations in breathing synchrony

Along with individual entropy rate and pairwise synchrony measured using PLV, we explored higher order effects among audience members (as triplets). We used two complementary information-theoretic measures to capture this higher-order interdependencies[3], namely Σ and Ω information. Σ information has been shown to capture higher-order structural correlations and is related to the Tononi-Sporns-Edelman (TSE) Complexity[4]. Ω-information captures the balance of redundancy and synergy among a group of variables and is equivalent to the more commonly known interaction information[5] for the case of triplets. We used the framework of multivariate auto-regressive (MVAR) model to infer Ω - *information* and Σ - *information* for triplets of participants. This framework is especially designed for oscillatory signals[6]. Average, Ω and Σ information was estimated for each participant by averaging over all triplets involving the participant.

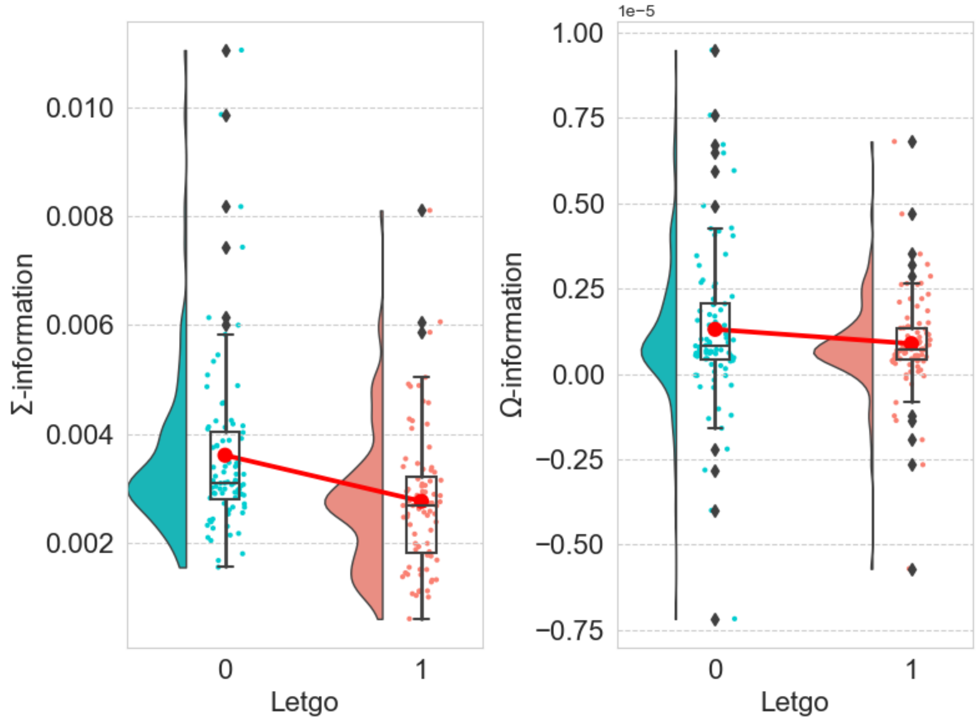

Figure S9. Higher order differences between strict and Letgo modes using triplet level (a) Σ-information and (b) Ω-information

Fig. S9 shows that Σ-information, decreased during *Let-go* performances ($\beta = -0.47$, $t_{40} = -3.43$, $p = 0.001$). Whereas, no significant change was observed for Ω-information ($\beta = -0.20$, $t_{40} = -1.36$, $p = 0.216$).

C.4 Effect of audience's vision

Fig. S10(a) shows a comparison of physical activity power spectra between the blindfolded and non-blindfolded audience members. Blindfolded audience members tended to show less physical activity than those who could see the performance, but the differences were not significant. Fig. S10(b) and (c) show comparison of P-A sync and A-A sync between the audience's sight type, respectively. For A-A sync, blindfolded audience members showed higher level of synchrony in both shorter and longer time scales. Similar tendency was also observed in P-A sync, but the difference was not significant. Fig. S10(d) and (e) show comparison of temporal variability in P-A sync and A-A sync between the audience's sight type, respectively. No significant effect of sight types was observed.

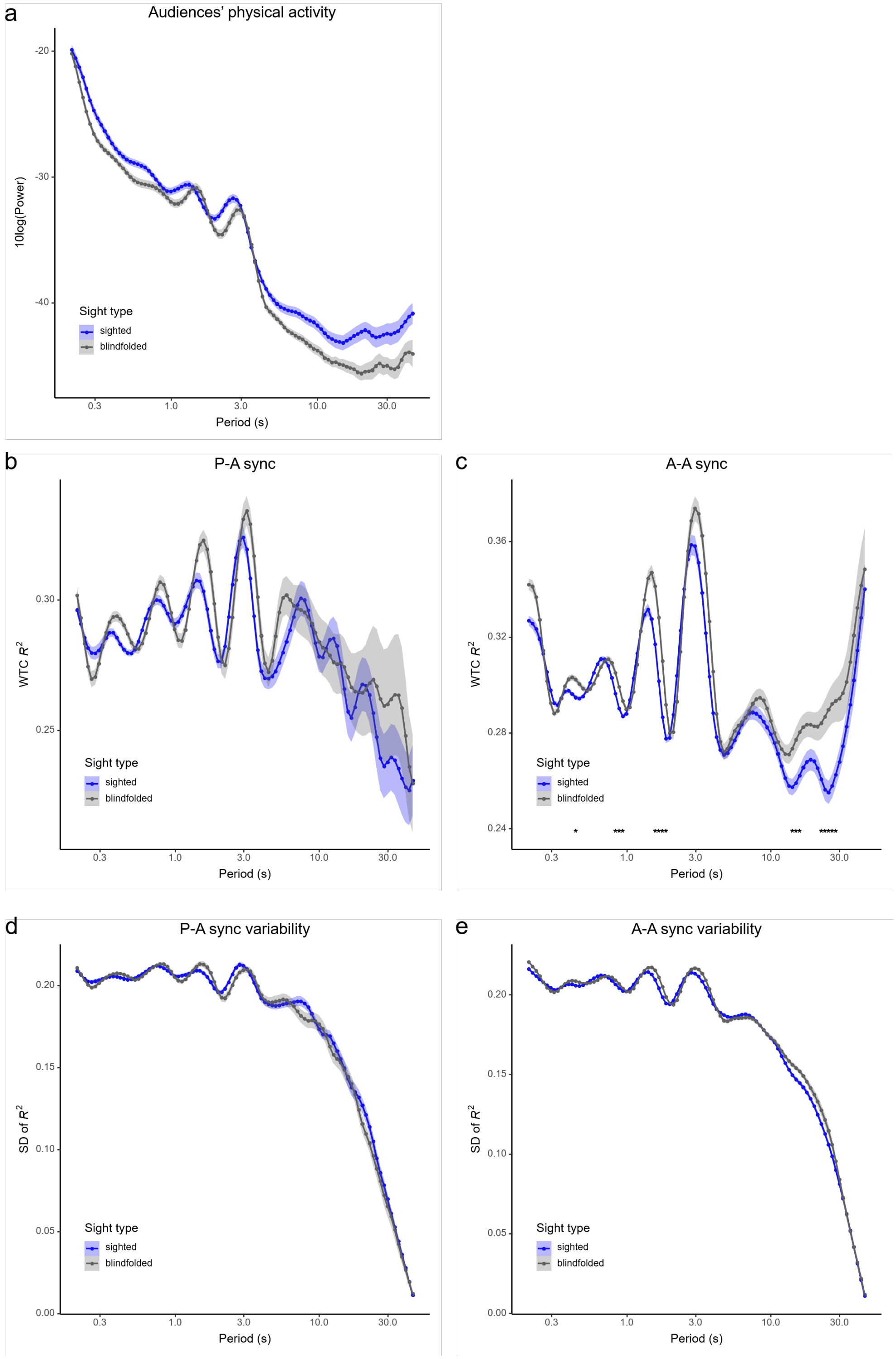

Figure S10. (a) Mean power spectra of the audience's physical activity of the two groups, comparing the effect of wearing a blindfold. Blindfolded audience show slightly less movement. (b) Mean P-A sync over different timescales (periods) for the two groups. (c) Mean A-A sync over different timescales (periods) for the two groups. Shaded areas indicate SEM over the four performers. Periods with significant difference between the sighted and blindfolded groups are marked by asterisks. *: p <0.05; FDR-corrected.

D Relationship between psychology and movement

D.1 Analysis by composition

When separating the performances by the compositions, the analysis of the correlation between subjective ratings and movement synchrony at different timescales revealed stronger correlations and anti-correlations for the performances of Haydn's composition (Table S4) than Mozart's. On the other hand, for the performances of Mozart's composition, the correlations were negligible (Table S5). These further supports the musicians' assessment of the performance itself, with the modes of performance being more strongly differentiated in the piece by Haydn than the piece by Mozart.

	**Beat-sync**			**Music-sync**			**Music-sync variability**		
**Rating**	r	t_{41}	p	r	t_{41}	p	r	t_{41}	p
				**P-A sync**					
PC1	**−0.40**	**−2.82**	**0.007**	**0.45**	**3.27**	**0.002**	**0.31**	**2.10**	**0.042**
Improvisatory	**−0.47**	**−3.44**	**0.001**	**0.43**	**3.06**	**0.004**	**0.46**	**3.30**	**0.002**
Innovative	**−0.32**	**−2.18**	**0.035**	**0.51**	**3.75**	**<0.001**	**0.42**	**3.00**	**0.005**
RiskTaking	**−0.50**	**−3.65**	**<0.001**	**0.47**	**3.40**	**0.001**	**0.43**	**3.03**	**0.004**
Engaging	**−0.31**	**−2.08**	**0.044**	0.23	1.50	0.141	0.19	1.26	0.216
Convincing	0.10	0.66	0.511	0.09	0.61	0.548	0.07	0.42	0.675
Familiar	−0.03	−0.19	0.848	0.25	1.62	0.112	0.21	1.36	0.182
Sleepy	−0.09	−0.57	0.569	−0.22	−1.48	0.147	0.08	0.49	0.629
				**A-A sync**					
PC1	−0.26	−1.73	0.090	**0.45**	**3.23**	**0.002**	**0.49**	**3.57**	**<0.001**
Improvisatory	**−0.33**	**−2.22**	**0.032**	**0.51**	**3.79**	**<0.001**	**0.57**	**4.43**	**<0.001**
Innovative	−0.24	−1.57	0.125	**0.44**	**3.18**	**0.003**	**0.57**	**4.43**	**<0.001**
RiskTaking	**−0.33**	**−2.25**	**0.030**	**0.47**	**3.38**	**0.002**	**0.53**	**4.01**	**<0.001**
Engaging	−0.29	−1.91	0.063	0.27	1.76	0.085	0.11	0.73	0.472
Convincing	0.27	1.77	0.085	−0.05	−0.35	0.727	−0.08	−0.52	0.609
Familiar	−0.26	−1.73	0.091	0.20	1.29	0.203	0.22	1.43	0.160
Sleepy	0.01	0.08	0.938	−0.03	−0.19	0.853	0.09	0.56	0.576

Table S4. Correlation between audience ratings and mean movement synchrony and its temporal variability, with only the two pieces composed by Haydn.

As an example, Fig. S11 illustrates the correlations between the first principal component of the audience ratings (PC1) and P-A sync in shorter and longer timescales as well as the temporal variability of the P-A sync in the longer timescales for the pieces of Haydn's composition.

	Beat-sync			Music-sync			Music-sync variability		
**Rating**	r	t_{41}	p	r	t_{41}	p	r	t_{41}	p
				**P-A sync**					
PC1	−0.11	−0.69	0.496	0.04	0.25	0.808	0.11	0.71	0.481
Improvisatory	−0.11	−0.70	0.489	0.05	0.31	0.759	0.01	0.09	0.927
Innovative	−0.04	−0.28	0.780	0.04	0.29	0.775	0.16	1.03	0.307
RiskTaking	−0.10	−0.66	0.513	0.05	0.34	0.734	0.20	1.29	0.205
Engaging	−0.12	−0.77	0.444	0.06	0.38	0.703	0.19	1.21	0.233
Convincing	−0.01	−0.04	0.970	−0.09	−0.56	0.576	0.17	1.08	0.285
Familiar	**0.35**	**2.42**	**0.020**	**−0.32**	**−2.16**	**0.037**	**−0.40**	**−2.81**	**0.008**
Sleepy	0.14	0.92	0.362	−0.11	−0.68	0.500	−0.18	−1.16	0.253
				**A-A sync**					
PC1	0.15	0.96	0.344	—	—	—	0.00	−0.03	0.977
Improvisatory	0.09	0.61	0.545	—	—	—	−0.11	−0.73	0.468
Innovative	0.18	1.16	0.251	—	—	—	−0.15	−0.98	0.335
RiskTaking	0.16	1.01	0.320	—	—	—	−0.06	−0.38	0.704
Engaging	0.09	0.57	0.571	—	—	—	0.05	0.32	0.752
Convincing	0.03	0.84	0.405	—	—	—	0.03	0.19	0.847
Familiar	0.25	1.68	0.101	—	—	—	−0.25	−1.62	0.112
Sleepy	0.16	1.01	0.316	—	—	—	−0.22	−1.41	0.165

Table S5. Correlation between audience ratings and mean movement synchrony and its temporal variability, with only the two pieces composed by Mozart. Remark: For the movement synchrony between audience (A-A sync), there were no periods of interest (timescales) where the performance modes showed significant effect of *Let-go* > *Strict* on the average synchrony (music sync).

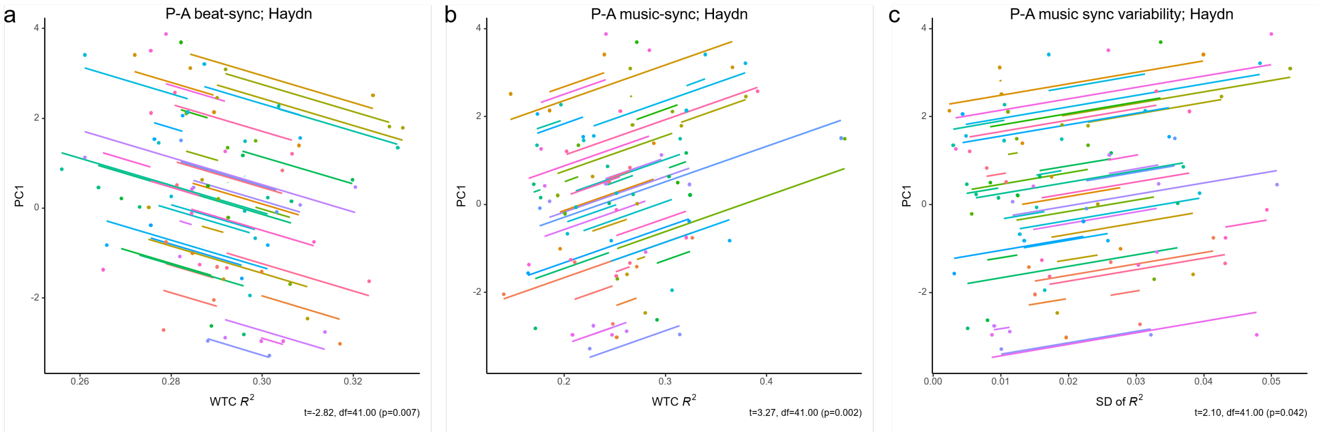

Figure S11. Relationship between the mean P-A sync in the shorter timescales (‘beat-sync’; a), in the longer timescales (‘music-sync’; b) and P-A sync temporal variability in the longer timescales (‘music-sync variability’) and the audience members’ perception (PC1) for Haydn’s pieces. Coloured points represent the two performances with the different modes for each subject. Coloured lines indicate the best linear fit of the relationship between the synchrony and the ratings for each subject, estimated using the multilevel models with the same slope (fixed effect) and varying intercepts (random effect).

References

1. Tellegen, G., Auke; Atkinson. Openness to absorbing and self-altering experiences ("absorption"), a trait related to hypnotic susceptibility. *J. Abnorm. Psychol.* **83**, 268–277, DOI: https://doi.org/10.1037/h0036681 (1974).

2. Liu, Y., San Liang, X. & Weisberg, R. H. Rectification of the bias in the wavelet power spectrum. *J Atmos Ocean. Technol* **24**, 2093–2102, DOI: http://doi.org/0.1175/2007JTECHO511.1 (2007).

3. Rosas, F. E., Mediano, P. A., Gastpar, M. & Jensen, H. J. Quantifying high-order interdependencies via multivariate extensions of the mutual information. *Phys. Rev. E* **100**, 032305, DOI: http://doi.org/0.1103/PhysRevE.100.032305 (2019).

4. Tononi, G., Sporns, O. & Edelman, G. M. A measure for brain complexity: relating functional segregation and integration in the nervous system. *Proc. Natl. Acad. Sci.* **91**, 5033–5037 (1994).

5. McGill, W. Multivariate information transmission. *Transactions IRE Prof. Group on Inf. Theory* **4**, 93–111 (1954).

6. Antonacci, Y. *et al.* Measuring high-order interactions in rhythmic processes through multivariate spectral information decomposition. *IEEE Access* **9**, 149486–149505 (2021).
